# Identification of Potential Pteridin Reductase-1 Inhibitors for the Treatment of Leishmaniasis: A Bioinformatics Approach

**DOI:** 10.3390/ph18081237

**Published:** 2025-08-21

**Authors:** Paulo R. da S. R. Júnior, Lúcio R. de Lima, Luciane B. Silva, Ryan S. Ramos, Vitor H. da S. Sanches, Njogu M. Kimani, Gustavo H. G. Trossini, Joaquín M. Campos, Cleison C. Lobato, Cleydson B. R. Santos

**Affiliations:** 1 Graduate Program in Pharmaceutical Sciences, Federal University of Amapá, Macapá 68902-280, AP, Brazil; paulorsrj2011@gmail.com (P.R.d.S.R.J.); cleison@unifap.br (C.C.L.); 2 Laboratory of Modeling and Computational Chemistry, Department of Biological and Health Sciences, Federal University of Amapá, Macapá 68902-280, AP, Brazil; luciorolima@gmail.com (L.R.d.L.); luciaanebarros@hotmail.com (L.B.S.); ryanquimico@hotmail.com (R.S.R.); the.chemical.vh@gmail.com (V.H.d.S.S.); 3 Natural Product Chemistry and Computational Drug Discovery Laboratory, Department of Physical Sciences, University of Embu, Embu P.O. Box 6-60100, Kenya; njogu.mark@embuni.ac.ke; 4 Laboratory of Integration Techniques Experimental and Computational in Drug Design, Department of Pharmacy, School of Pharmaceutical Sciences, University of São Paulo, São Paulo 05508-900, SP, Brazil; trossini@usp.br; 5 Department of Pharmaceutical and Organic Chemistry, Faculty of Pharmacy, Campus of Cartuja, University of Granada, 18071 Granada, Spain; jmcampos@ugr.es

**Keywords:** *Leishmania major*, pteridine reductase-1, virtual screening, methotrexate, molecular docking

## Abstract

**Background/Objectives:** Leishmaniasis is an infectious disease caused by digenetic protozoa of the genus *Leishmania*, transmitted by infected female sandflies of the *Phlebotominae* subfamily. Current treatments are limited, relying on drugs that were not specifically developed for this disease and are often associated with high toxicity and elevated costs. Among alternative therapeutic strategies, antifolate compounds have been investigated due to their ability to inhibit dihydrofolate reductase (DHFR), an enzyme essential for folate metabolism in the parasite. However, the parasite circumvents DHFR inhibition through the activity of pteridine reductase-1 (PTR-1), which maintains folate reduction and ensures parasite survival. In this context, this study aimed to identify potential PTR-1 inhibitors in *Leishmania major* through in silico approaches. **Methods:** The methodology included virtual screening of molecular databases, Tanimoto similarity analysis, pharmacokinetic and toxicological predictions, and biological activity evaluation in silico. The most promising compounds were further analyzed via molecular docking. **Results:** The virtual screening resulted in 474 molecules, of which 4 structures (M601, M692, M700, and M703) showed high potential as PTR-1 inhibitors in *Leishmania major* throughout all stages of the methodology employed, especially in the results of molecular docking where they exhibited strong binding affinities and significant interactions with key residues of the target enzymes. **Conclusions:** This work provides a solid foundation for advancing these molecules into experimental validation, contributing to the development of safer and more effective therapeutic alternatives for the treatment of leishmaniasis.

## 1. Introduction

Leishmaniasis is an infectious disease caused by parasites of the genus Leishmania, transmitted by the female sandfly of the phlebotomine subfamily and is present in almost 100 countries [1]. Despite being a neglected tropical disease, it has gained significant attention due to its disproportionate impact on underdeveloped nations and highly vulnerable populations. In Brazil, the incidence of leishmaniasis cases has risen exponentially, particularly in the northern region. This surge could be attributed to deforestation, rapid urbanization, and extensive construction activities, which have significantly disrupted the habitat of the sandfly vector [2,3].

The disease has several clinical forms grouped into four categories that vary according to the species involved and the parasite–host relationship. They include visceral leishmaniasis, cutaneous leishmaniasis, mucocutaneous leishmaniasis, and diffuse cutaneous leishmaniasis [4]. Cutaneous leishmaniasis (CL) is the most prevalent form of leishmaniasis, characterized by skin lesions, mainly ulcers, on exposed body parts. These lesions leave lifelong scars, causing significant disability, stigma, and a reduced quality of life. Around 95% of CL cases occur in the Americas, the Mediterranean basin, the Middle East, and Central Asia [5]. In 2020, more than 85% of new cases of CL occurred in 10 countries: Afghanistan, Algeria, Brazil, Colombia, Iraq, Libya, Pakistan, Peru, Syria, and Tunisia. Globally, it is estimated that between 700,000 and 1 million new cases occur annually [6].

Parasites of the genus Leishmania are obligatory intracellular parasites that affect cells of the mononuclear phagocytic system, thus hindering the immune response. They have two main morphological characteristics: a flagellated promastigote, which can be found in the digestive tract of the insect vector, and a non-flagellated amastigote form, found in the mammalian host [7]. The first line of treatment approved by the Brazilian Ministry of Health consists of pentavalent antimonials. However, these drugs have low efficacy rates and high toxicity, therefore raising concerns about their effectiveness. Other drugs also make up the therapeutic regimen according to the species of agent involved, such as Amphotericin B, which, despite showing good efficacy, also has high toxicity and is costly [2,8]. The parenteral form of administration of these drugs, which is considered invasive, makes it difficult for patients to adhere to treatment and hence contributes to the development of drug-resistant infectious forms [9].

Antifolate drugs have been extensively researched for treating protozoal diseases with emphasis on malaria, but their efficacy against leishmaniasis has been limited due to the parasite’s developing resistance. Since the main therapeutic target in these protozoa is a folate-reducing enzyme called dihydrofolate reductase (DHFR) [10], in the genus Leishmania, this strategy has proved to be ineffective due to an enzyme called pteridine reductase-1 (PTR-1), which has the function of reducing free or conjugated pteridines for its development. However, PTR-1 is also capable of reducing folates to a lesser extent, but enough to compensate for the suppression of DHFR, thus resisting the action of antifolates [11,12]. Since the parasite cannot survive the inhibition of these two enzymes, it is worth developing an antagonist that acts in conjunction with a DHFR-thymidylate synthase (TS) antifolate drug or that acts simultaneously on both enzymes since the sites of action have similar characteristics [12,13].

Given the folate reduction pathway’s potential as a drug target for leishmaniasis, this study aimed to discover molecules with potential inhibitory activity against PTR-1, a key enzyme in *Leishmania major*. A pharmacophoric model was constructed as a reference for virtual screening of molecular libraries, followed by in silico evaluations of pharmacokinetic profiles and structure–activity relationships; thus, it was possible to obtain four structures with a good probability of antiprotozoal activity and with lower toxicological risk. The results obtained may motivate in vivo biological activity assays, including in association with DHFR inhibitors, aiming at a more effective therapy.

**Scheme 1:**
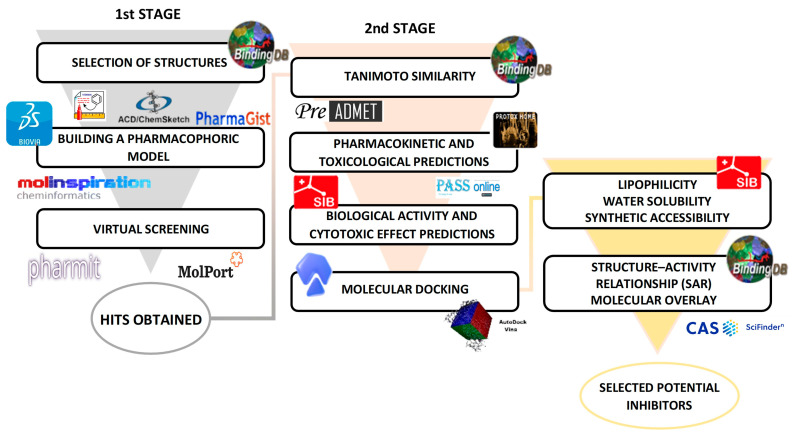
Main methodological steps.

The methodological approach is outlined in Scheme 1 (for more details, see the Section 3).

## 2. Results and Discussion

### 2.1. Pharmacophoric Model Generation

**Figure 1:**
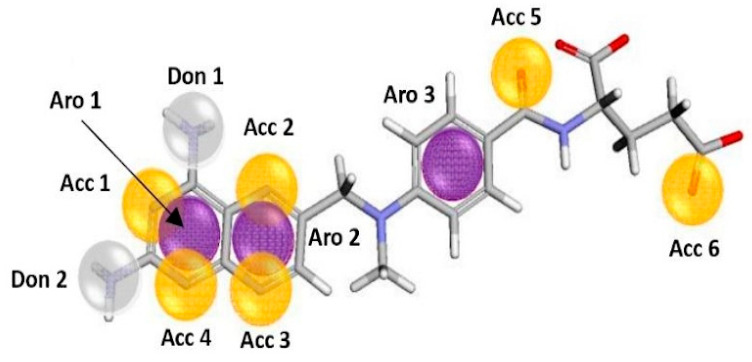
Pharmacophoric characteristics, obtained from alignment by the Pharmagist online server (yellow sphere: Acc; purple sphere: Aro; white sphere: Don).

The pharmacophore model was developed from selected structures, using the Pharmagist online server (https://bioinfo3d.cs.tau.ac.il/PharmaGist, accessed on 2 February 2024). It was based on the alignment of the reference structure with the other structures in the ligand group. From the results, the alignment with the best score was selected, which was 72.746 (Table 1), a value considered to be high for alignment [14]. This is probably due to the presence of the pteridine ring and benzoic acid in all the structures selected. Figure 1 shows the qualitative characteristics of the best-selected model.

### 2.2. Virtual Screening Based on Pharmacophore

On the Pharmit online platform (https://pharmit.csb.pitt.edu/, accessed on 2 February 2024), Molport was selected to carry out pharmacophore-based virtual screening (VS) to obtain molecules (HITS) within the pharmacophore characteristics obtained on Pharmagist. As a strategy for better refinement of these structures, the maximum and minimum values of the physicochemical characteristics of the nine reference structures were used as a selection filter (Table 2).

The 11 pharmacophoric characteristics were not enough to obtain the VS hits on the Pharmit platform, so it was necessary to create pharmacophoric hypotheses according to the methodological strategy adopted by Ferreira et al. [15].

The number of combinations was obtained using Equation (1) (below), where C = number of combinations; *p* = type of model (*p* ≠ 0, *p* = 1, *p* =2, …, *p* = ∞), and *n* = number of variables in the pharmacophoric model. Equation (1):

(1) $$C_{p,n}=\;\frac{n!}{p!\left(n-p\right)!}$$

Considering the number of variables *n* = 11, we took into account the studies by Dube et al. [16] and the contributions of the studies by Phadke [17], which showed that the main pharmacophore characteristics involved in the inhibitor-PTR-1 interaction are two H-bond donors, a hydrophobic aromatic characteristic and an aromatic ring characteristic, so the characteristics Don 1, Don 2, Aro 1 and Aro 2 were excluded from the set of variables, making it *n* = 7.

The possible combinations with seven variables were again submitted to hit searches on the Pharmit platform, analyzed by simple combination and without repetitions, resulting in a total of 474 molecules.

### 2.3. Tanimoto Similarity

Based on the assumption that “structurally similar compounds have similar physicochemical properties and possibly similar biological profiles” [18], a total of 474 preliminary hits were analyzed on the BindingDB web server for Tanimoto similarity studies. Molecules that exhibited a similarity index greater than or equal to 0.6 (60% similarity) with respect to the reference structure methotrexate (MTX) were selected. This cutoff point was defined based on the findings by Wang et al. [19], where they observed, through target fishing, that a Tanimoto similarity coefficient above 0.5 is considered an appropriate threshold to exclude irrelevant targets or false positives. The default threshold of the pharmaceutical target seeker (PTS) tool, which predicts targets for a molecule by overlaying its structure with 3D ligand structures of macromolecular targets, was also taken into consideration. Values below 0.6 indicate low similarity [20]. The process resulted in a total of 23 hits.

### 2.4. Pharmacokinetic and Toxicological Predictions

The 23 hits obtained from the Tanimoto similarity analysis were subjected to pharmacokinetic and toxicological predictions using the online server PreADMET. First, pharmacokinetic predictions were performed, evaluating absorption criteria such as human intestinal absorption (HIA), Caco-2 cell permeability, MDCK (Madin-Darby canine kidney) cell permeability, as well as distribution factors such as plasma protein binding (PPB) and blood–brain barrier (BBB) permeation. The MTX was used as a control in the selection of hits with a better pharmacokinetic profile. Additionally, commercially available drugs such as meglumine antimoniate (MGL), amphotericin B (ANF-B), and pentamidine (PTM), which are standard medications for the treatment of leishmaniasis recommended by the Brazilian Ministry of Health, were also used to contribute to the selection of desirable hits. The most active molecule from the reference structures group, BDBM50398391 (BDB-1), was added for comparison purposes.

In pharmacokinetic studies, HIA is an important criterion when it is considered that the drug must be administered orally; therefore, acceptable results must present values above 70% [21]. The reference compound (MTX) exhibited a value of 36.6%. Hits that presented higher values were considered acceptable, with particular attention given to hits MolPort-008-010-692, MolPort-008-010-700, and MolPort-008-010-703, which showed an average of 95% similarity.

Conversely, the drugs MGL and ANF-B showed low values (2.7% and 4.7%, respectively), justifying their parenteral administration. However, PTM showed 86% absorption, like the most active molecule (BDB-1), which exhibited 83% absorption. For Caco-2 cell permeability predictions (nm/sec), the classification is as follows: low permeability < 4 nm/s, medium permeability between 4 and 70 nm/sec, and high permeability > 70 nm/s [22]. Permeability in MDCK cells (nm/s) was also evaluated, using the following classification: low (<25 nm/s), medium (25–500 nm/s), and high (>500 nm/s) [23].

MTX displayed an average absorption value of 18.88 nm/s in Caco-2 cells, which is close to the value exhibited by the molecule BDB-1 (18.26 nm/s). Therefore, all hits with medium and high permeability results were considered acceptable. In MDCK absorption, MTX showed low permeability (2.44 nm/s), as did BDB-1 (1.63 nm/s). Since the majority of the hits also presented low values, this criterion was not used as an exclusion factor.

In drug design, considering the percentage of plasma protein binding (PPB) is one important parameter in optimizing drug efficacy and safety. Knowing the plasma protein binding profile of a drug allows predicting its distribution in target tissues, its systemic exposure, the need for dose adjustments, and potential interactions with other drugs that may compete for protein binding [24].

The PreADMET classifies a high binding rate as %PPB > 90%. The drug MTX exhibited a 44.5% protein binding rate. Considering that MTX is an anti-folate with a toxic nature due to its inhibition of human DHFR and serves as a reference in this research, hits with a percentage ≥ 44.5 were accepted in order to obtain a variability of structures with medium and high binding rates, as observed in the results of MGL, which showed a high %PPB (96%), and ANF-B, with an average %PPB rate (39%).

Penetration of the blood–brain barrier (Cbrain/Cblood) is an important parameter in the development of drugs as it is related to the action of compounds in the central nervous system. For this research, which aims to obtain leishmanicidal agents, this value must be less than 1 (Cbrain/Cblood < 1) since any value above is an indication that the compound is in high concentration both in the blood and in the brain, which may cause adverse effects [25]. Within the previous descriptors, only 18 molecules were selected (Table 3).

Toxicological predictions were also performed on the PreADMET online server, and the toxicological class and lethal dose (LD_50_) predictions were performed on the Protox II server. By predicting the toxicological class and lethal dose of a drug candidate, researchers can identify substances that have an acceptable safety profile and promise for clinical development. This assists in selecting safer candidates, optimizing formulations, determining appropriate therapeutic doses, and minimizing risks associated with drug use. In addition, the prediction of toxicity and lethal dose contributes to compliance with the regulatory and ethical requirements necessary for the approval and commercialization of a drug.

Predictions of carcinogenicity in rats and mice were performed, where a “negative” result indicates a carcinogenic profile and a “positive” result indicates a non-carcinogenic profile. The reference structure, MTX, exhibits a negative value, which was expected due to its mechanism of action. Therefore, hits that showed a positive result for rats, mice, or both were selected, as demonstrated by the results of ANF-B and PTM. The BDB-1 structure exhibited the same profile as the control drug, and for MGL, the analysis method was unable to provide results.

**Figure 2:**
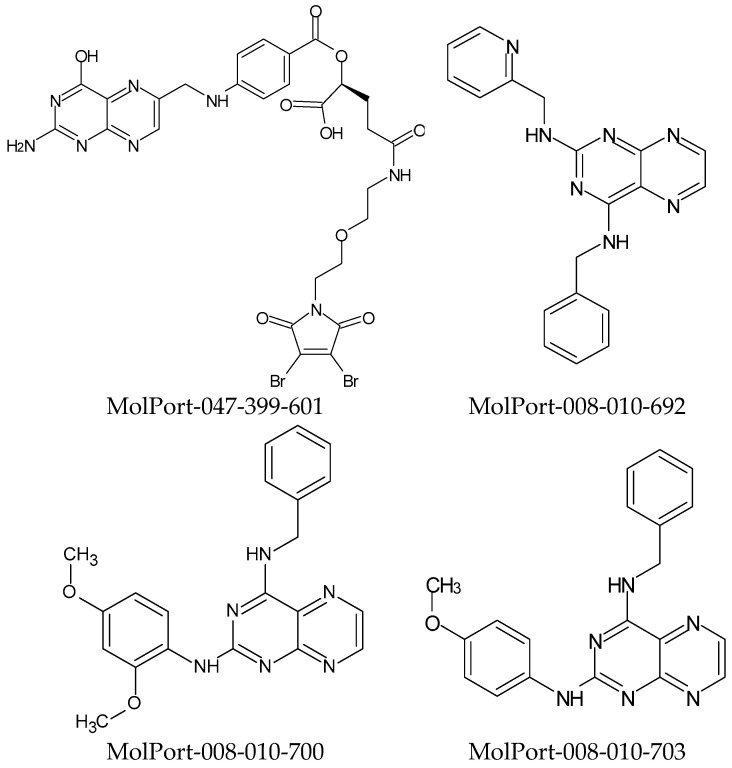
Selected hits from pharmacokinetic and toxicological predictions.

The Ames test is particularly sensitive to detecting point mutations, such as base pair substitution changes, and is widely used to assess the mutagenic potential of chemicals [26]. In PreADMET, it was possible to perform mutagenicity predictions, where it was observed that only two hits had non-mutagenic results (MolPort-008-010-700 and MolPort-008-010-703). The results of the Protox II server were quite satisfactory since the reference structure presented LD_50_ = 3 mg/Kg, categorized as toxicity class 1. The evaluated hits presented toxicity classes of between 3 and 5. Based on the results of carcinogenicity, mutagenicity, and LD_50_ predictions, four hits (Figure 2) were selected (Table 4).

### 2.5. Biological Activity and Cytotoxic Effect Predictions

Biological activity predictions play a crucial role in drug development. They provide valuable information about how a chemical interacts with specific molecular targets in the body and how it can affect relevant biological processes. The PASSonline server makes predictions about a wide range of biological activities, using a machine learning approach, to correlate the chemical structure of substances with their reported biological activities. Thus, using the results of the pivot structure as evaluative parameters, the four resulting hits were submitted to predictions of biological activity (Table 5).

The four hits analyzed, abbreviated as M601, M692, M700, and M703, produced satisfactory results for the selected parameters: folate antagonist, DHFR inhibitor, PTR-1 inhibitor, and antiprotozoal activity. They all showed possible inhibitory activity for PTR-1 (the target enzyme of the study) and folate antagonist, which indicates probable inhibitory activity for the DHFR and TS enzymes. The antiprotozoal parameter was also highlighted in the results, except for structure M601, all of which showed anti-trypanosome activity, which may represent a starting point for the future development of inhibitors of the pteridine metabolism of other trypanosomatids [27,28].

Cytotoxicity predictions allow for the evaluation of the potential of a substance to cause damage or adverse effects to healthy cells in the body. In PASSonline, the server uses information about the chemical structure of the substance in question to compare it with a database containing information on compounds with known cytotoxic activity. The algorithm analyzes the structural similarity between the substance under evaluation and the reference compounds, considering characteristics such as functional groups, molecular conformation, and other relevant properties [29].

The four molecules were submitted for analysis and demonstrated cytotoxic activity potential. Molecules M692, M700, and M703 showed tumor activity in bone and colon tissues, while molecule M601 showed activity in a larger number of tissues: brain, pancreas, skin, and hematopoietic/lymphoid tissue (Table 6)

### 2.6. Molecular Docking

Molecular docking is an important tool in drug design as it plays a key role in understanding the interaction between a drug candidate molecule and its biological target. In this study, the docking tests were performed in the AutoDock-Vina software in the PYRX version 0.8 graphical interface. First, the validation of the docking method was carried out [30], also known as re-docking, to evaluate the ability of the method to reproduce the orientation and binding mode of the experimental crystallographic pose, calculating the root-mean-square deviation (RMSD) of the poses of the theoretical binders with the experimental ones. The acceptable RMSD values are below 2.0 Å [31].

**Figure 3:**
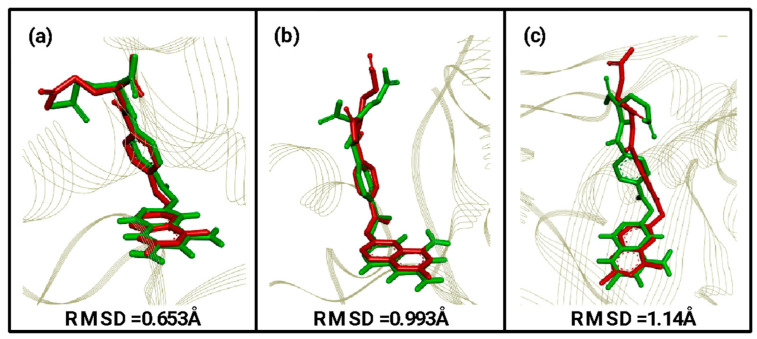
RMSD values obtained through validation of the molecular docking method with their respective overlapping of theoretical (green) and computational (red) ligands. (**a**) MTX-1DLS, (**b**) MTX-1E7W, and (**c**) MTX-5X66.

The results obtained were: MTX-1DLS complex, RMSD = 0.653 Å; MTX-1E7W, RMSD = 0.993 Å and MTX-5X66, RMSD= 1.14 Å (Figure 3). With all values below 2 Å, the docking method used for this study was considered valid.

Docking studies were carried out with the four promising structures: M692, M700, M703, and M601, with the PDBs selected targets for this research (1DLS, 1E7W, and 5X66).

**Figure 4:**
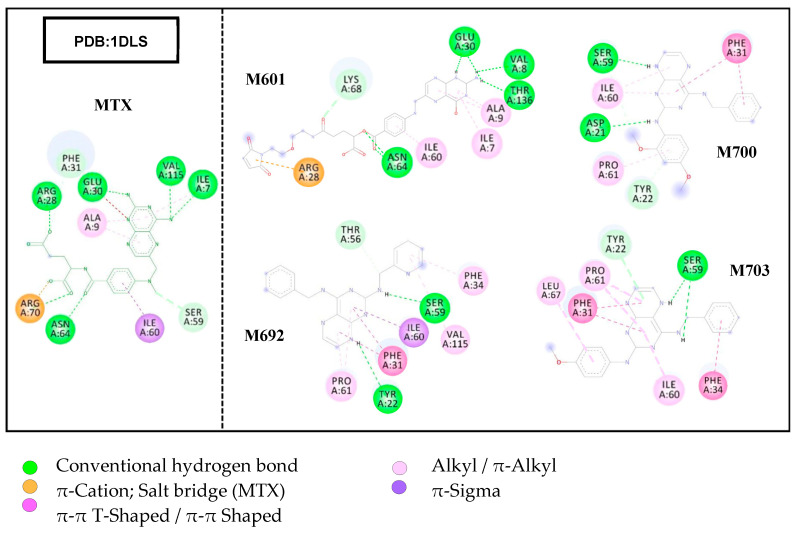
Results of molecular docking assays showing interactions with amino acid residues of the DHFR target site.

The molecular docking method used identified the interactions with the amino acid residues of the active site of the PDB 1DLS protein complexed with MTX, a competitive inhibitor. Interactions with common hydrogen bonds were observed around the β-sheet, with the amino acids ILE-7 and VAL-115, in the α-helix with the amino acids GLU-30, ARG-28, and ASN-64, and in the loop with ARG-70. Hydrophobic interactions of the π-alkyl type were observed in the β-sheet with ALA-9 and ILE-7, of the π-sigma type in α-helix with ILE-60. PHE-31 interacts with the Van der Waals force also in the α-helix. Figure 4 shows the result of the docking with the four promising structures.

The results of the docking assays showed that the M601 ligand presented the highest number of interactions with the same amino acid residues that interacted with MTX in the DHFR target, as well as the lowest binding affinity energy, ΔG = −9.6 Kcal/mol, followed by the M692 ligand that presented four interactions with the residues involved in the MTX/DHFR complex, with the second lowest value of ΔG = −9.2 Kcal/mol. Ligands M700 and M703 showed fewer interactions but presented acceptable ΔG values since the ligand M700 resulted in the highest value of all ΔG= −8.8 Kcal/mol.

**Figure 5:**
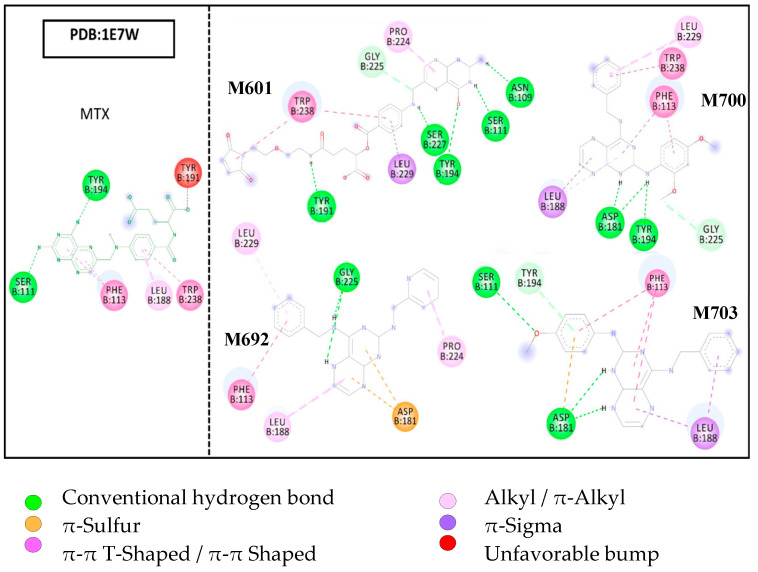
Molecular docking results, evidencing the interactions with the amino acid residues of the PTR-1 target site.

The crystallographic structure of pteridine reductase complexed with MTX, at a resolution of 1.75 Å, was selected for molecular docking studies. The results showed two hydrogen bonding interactions with the amino acid residues TYR194 in the α-helix and SER111 in the loop. PHE113, in a loop, showed hydrophobic interactions of the π-π stacked type with the pteridine rings. On the aromatic ring, there were interactions with residues LEU188, of the π-alkyl loop type, and with TRP238, of the π-π T-shaped type in α-helix. An unfavorable interaction was observed with the TYR191 residue in the loop region. Figure 5 illustrates the molecular docking results of the studied structures.

The results of docking the ligands with PDB 1E7W showed considerable interactions with amino acid residues related to the 1E7W/MTX complex. Molecule M601 showed nine interactions with residues of the target active site, five of which are of a hydrogen bond type. Three of these hydrogen bonds are with the residues SER111, TYR191, and TRP238 (1E7W/MTX). The M692 ligand showed six interactions in total, two with the related residues of the 1E7W/MTX complex, which are π-π shaped hydrophobic with PHE113 and π-alkyl with LEU188.

The results also showed that the M700 ligand interacted with three described residues of the reference complex; PHE113 with two interactions (π-π T-shaped and π-stacked), LEU188 also with two interactions (π-alkyl and π-Sigma), and a hydrogen bond-type interaction with TYR194. Ligand M703 showed five interactions in total, of which four are with related residues of the complex, SER111 with hydrogen bonding, PHE113 with π-π T-shaped and π-stacked, LEU188 with π-sigma, and TYR194 with π-donor interaction hydrogen.

In the absence of crystallographic structures of TS from leishmania, PDB 5X66 was used for molecular docking studies. Considering that the substrates used by the human TS enzyme are the same used by the parasite’s TS, it becomes relevant to evaluate the interactions of the resulting ligands to obtain possibly more selective inhibitors.

**Figure 6:**
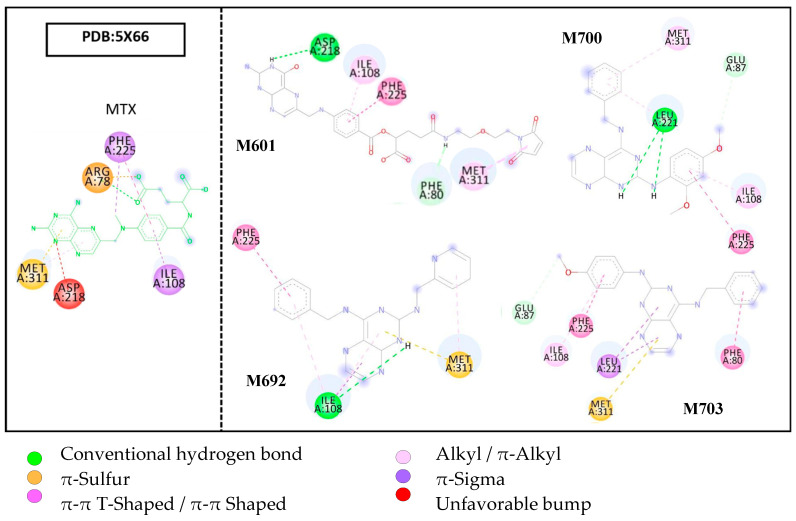
Molecular docking results show interactions with amino acid residues of the TS target site.

Molecular docking results of the crystallographic structure of the enzyme thymidylate synthase complexed with MTX showed few interactions with the active site despite the ligand structure being conjugated. An unfavorable acceptor–acceptor interaction was observed in the terminal portion of pteridine rings with the residue ASP218 in the β-sheet. The loop MET311 showed two interactions, π-sulfuric and π-alkyl. Residue ILE108 showed a looped π-sigma interaction. In α-helix, the residue PHE225 presented two interactions, π-π T-shaped and π-sigma. The residue ARG78 in the loop also presented two interactions, a hydrogen bond and π-sulfuric. Figure 6 illustrates the molecular docking results of the structures studied.

Molecular docking assays of the ligands at the target site of the TS enzyme showed few interactions, but there was a relationship with the amino acid residues present in the 5X66/MTX complex. Ligand M601 showed five interactions, of which four were with the residues of the complex (ASP218–hydrogen bridge, ILE108–π-alkyl, MET311–π-alkyl, and PHE225–π-π stacked). Ligands M700 and M703 also interacted with the three referenced amino acid residues ILE108, MET311, and PHE225, all in a hydrophobic way.

Evaluating the binding affinity (∆G) energies of the ligands suggested in the molecular docking study, it was possible to observe that the MTX reference structure and the M601 ligand had higher energy (∆G = −6.6 kcal/mol) for the target PTR-1 (1E7W), showing little affinity. In contrast, the ligands M692 (∆G = −8.6 kcal/mol), M700 (∆G = −8.4 kcal/mol), and M703 (∆G = −8.0 kcal/mol) obtained lower ∆G values, thus conferring greater affinity for the target.

**Figure 7:**
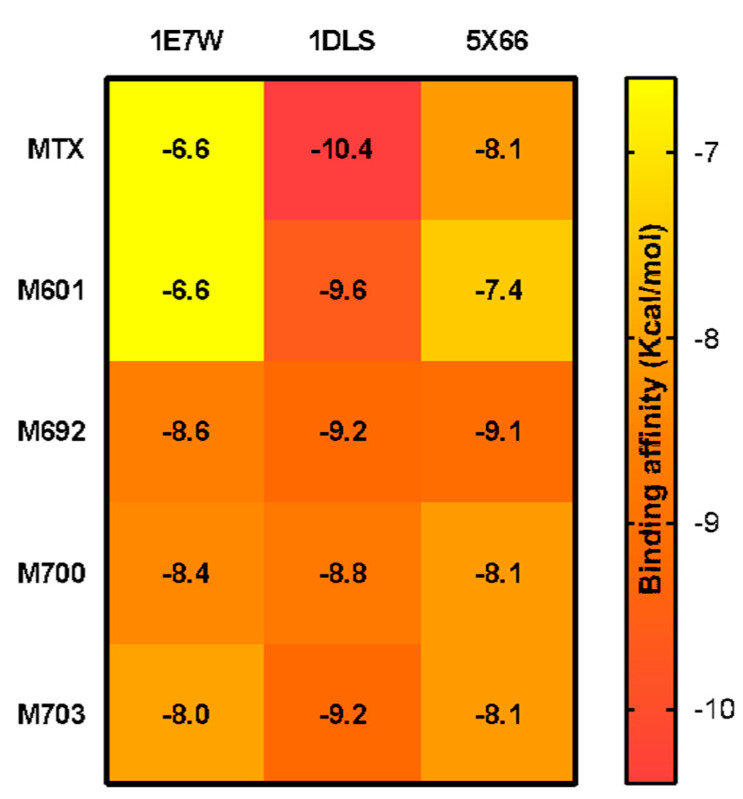
The heatmap graph of binding affinity values (∆G) of promising structures and the commercial drug MTX, at the active sites of PDBs 1E7W, 1DLS, and 5X66.

The results for the PDB 1DLS, the MTX reference structure, presented lower energy in relation to all the ligands (∆G = −10.4 kcal/mol), agreeing with the literature. However, they also presented values close to the reference (M601 ∆G = −9.6 kcal/mol, M692 ∆G = −9.2 kcal/mol, M700 ∆G = −8.8 kcal/mol, and M703 ∆G = −9.2 kcal/mol). The results of molecular docking of ligands in PDB 5X66 obtained promising values, especially ligand M692 (∆G = −9.1 kcal/mol), which presented lower energy than the MTX reference structure (∆G =−8.1 kcal /mol). Ligands M700 and M703 showed similar values ∆G =−8.1 kcal/mol, and ligand M601 showed the lowest affinity with ∆G = −7.4 kcal/mol (Figure 7).

It can be concluded that, based on the results obtained from molecular docking, the four ligands derived from the entire screening process showed affinity for the selected targets. Overall, it was observed that all ligands exhibited greater selectivity for DHFR (1DLS), which can be attributed to the choice of the reference structure (MTX) being more selective for DHFR. This validates the effectiveness of the proposed scientific methodology for this study.

The results also demonstrated the interactions of the ligands with the same amino acid residues involved with the reference inhibitor, MTX. Notably, M692 showed the highest affinity for all three targets, indicating a multitarget profile. Considering that multitarget drugs play an important role in the field of drug therapy, as they can broaden the spectrum of action and improve treatment effectiveness, reducing the need for combination with other drugs, it would be highly beneficial to develop an orally administered drug for a population that struggles with long-term treatment adherence. However, these results warrant further in-depth studies such as molecular dynamics simulations and in vitro assays.

### 2.7. Prediction of Lipophilicity and Water Solubility and Predicted Synthetic Accessibility (SA)

To evaluate the possibilities of future in vivo tests, it became important to determine the lipophilicity and water solubility predictions of promising compounds so that they could help in the preparation of experimental solutions. Predictions of synthetic accessibility were also made, which are an essential tool in the drug discovery process, helping guide the selection and development of candidate compounds in an efficient and economical way [30]. Promising molecules were analyzed through the SWISS-ADME web server, first with the reference (MTX) and with BDB-1 for comparison purposes.

The compounds (MTX) and BDB-1 showed the lowest consensus LogPo/w values (−1.09 and 0.52, respectively), indicating low lipophilicity. Among the promising molecules investigated in this study, structures M692, M700, and M703 showed higher consensus LogPo/w values (2.15, 2.90, and 2.88, respectively), which is a good indicator for drug candidate molecules (Table 7). This can be attributed to the presence of the pteridine ring in their chemical structures, which is an aromatic and hydrophobic group.

The values of the water solubility predictions corroborate the previous observations; the promising molecules were found to be moderately to poorly soluble in water, with LogS values ranging from −5.26 to −6.05 (Table 8). The estimate of water solubility is based on the qualitative LogS scale (ESOL, Ali, and SILICO-IT): highly soluble > 0 > very soluble > −2 > soluble > −4 > moderately soluble > −6 > slightly soluble > −10 > insoluble [32].

In the evaluation of the synthetic accessibility estimate, structures M692 and M703 showed an SA score of 2.84 and 2.85, respectively, while structures M601 and M700 showed a score of 4.75 and 3.09, respectively. Compared to the control compounds (MTX and BDB-1), the SA values were close, ranging from 2.85 to 4.75. Both structures (M692 and M703) were easy to synthesize, considering the parameters reported in the literature [33].

### 2.8. Structure–Activity Relationship (SAR) and Molecular Overlay of Promising Molecules

The identified promising molecules were searched in the SciFinder^®^ online database (https://scifinder.cas.org/, accessed on 23 March 2024) to obtain additional information regarding their biological activity. However, no specific data related to the biological effects of the selected compounds were found, aside from some physicochemical properties already reported in the MolPort database. Notably, the presence of the pteridine ring in these molecules is of particular interest, given its well-documented therapeutic potential [34].

Pteridines are aromatic heterocyclic compounds composed of fused pyrazine and pyrimidine rings. They are biosynthesized by a wide variety of living organisms, where they function as pigments, enzyme cofactors, or modulators of immune responses [35]. Due to their versatile chemical structure, pteridine derivatives have been extensively studied and are associated with a broad spectrum of biological activities, including antiviral, antifungal, antiparasitic, nitric oxide synthase inhibition, antitumor, anti-inflammatory, antibacterial, neuroprotective, antihypertensive, and anti-osteoporotic effects, among others [34]. The substantial evidence supporting the diverse biological activities of pteridine derivatives reinforces the relevance of the in silico findings obtained in this study, further justifying the progression to in vitro and in vivo validation of the selected compounds.

In addition, considering the structural similarity of the identified molecules to other bioactive pteridine-based compounds reported in the literature, there is a strong rationale to hypothesize that these candidates may exhibit not only inhibitory activity against PTR1 but also potential multitarget effects that could enhance their therapeutic efficacy against *Leishmania major*. This multifunctional profile is particularly advantageous in the context of neglected tropical diseases, where drug resistance and treatment limitations remain significant challenges. Therefore, further experimental studies, including biochemical assays and efficacy tests in parasitic models, are essential to validate the therapeutic potential of these compounds and to explore possible synergistic mechanisms of action.

Molecular overlap analysis is a valuable tool for predicting the binding affinity of candidate molecules to their biological targets. When a molecule exhibits similar steric and electrostatic properties to a reference drug, it is more likely to interact with the same amino acid residues at the active site, potentially resulting in comparable or even superior binding affinity [18]. In this study, the molecular structures of the four promising compounds were superimposed onto the commercial drug methotrexate (MTX) and the most active molecule in the studied series, BDB-1. The analysis considered varying electrostatic contributions at 25%, 50%, 75%, and 100%, as shown in Table 9.

The results revealed that molecules M601 and M703 exhibited the highest overlap values in relation to both MTX and BDB-1 across all contribution ratios, suggesting a strong similarity in their steric and electronic fields. This finding is particularly relevant since higher overlap values are generally associated with better accommodation within the enzyme’s active site, potentially translating into greater inhibitory activity. Notably, M703 showed the highest overlap with BDB-1 at 50% and 75% electrostatic contribution (0.49 and 0.44, respectively), indicating a balanced steric-electrostatic profile favorable for interaction with the target enzyme. These observations reinforce the potential of M601 and M703 as strong PTR1 inhibitors, supporting the progression to further computational and experimental validation steps, such as molecular dynamics simulations and in vitro enzyme inhibition assays.

**Figure 8:**
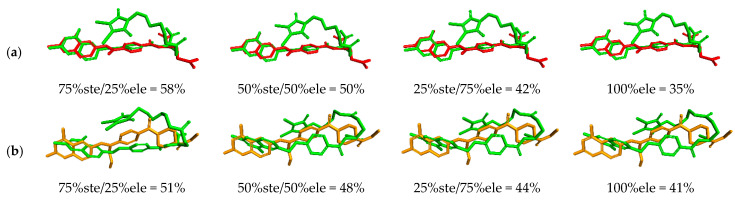
Similarity analysis according to the electronic overlap between the M601 molecule (green), the MTX structure (red (**a**)), and BDB-1 (orange (**b**)).

The results of the molecular overlay between the promising molecules and the MTX and BDB-1 structures showed that, for an electronic contribution of 25%, the range was 43% to 58%; for 50%, the range was 40% to 50%; for 75%, the structures ranged from 36% to 44% and for 100% electronic contribution, the range was 32% to 41%. The best overlap occurred between the M601 molecule and the MTX and BDB-1 structures (Figure 8).

## 3. Materials and Methods

### 3.1. Selection of Structures

**Figure 9:**
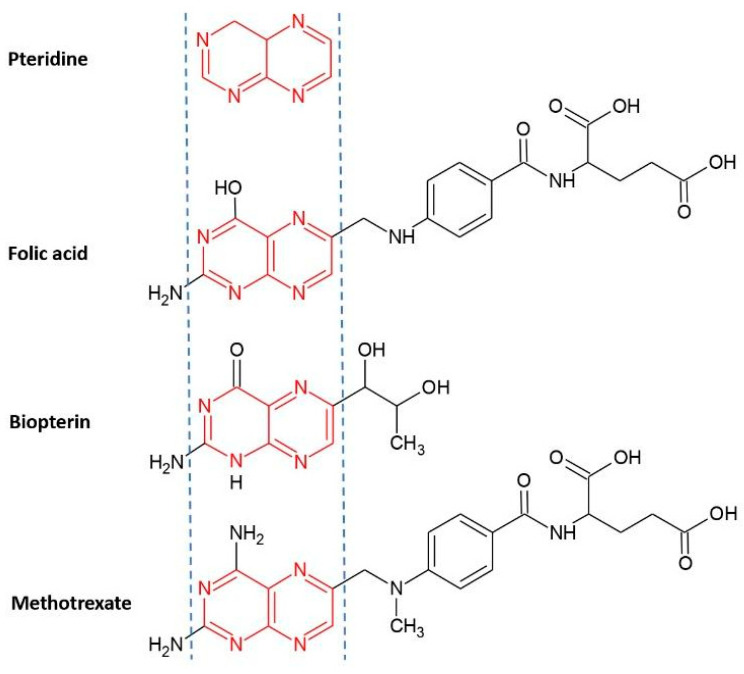
Chemical structure of pteridine present in the structure of folic acid, biopterin, and methotrexate.

The first step of the study was the selection in ascending order of Ki (inhibition constant) of 9 chemical structures with proven inhibitory activity for PTR-1 and DHFR enzymes from *Leishmania major* hosted in the online database BindingDB (https://www.bindingdb.org, accessed on 12 January 2024). The commercial drug methotrexate (4-amino-N10 methyl pteroglutamic acid, MTX) was used as a reference structure for all stages of the project due to its inhibitory activity for PTR-1 and DHFR of *L. major* already determined in the literature [12]. As a selection criterion, molecules that contained a pteridine ring in their structures were selected due to the affinity of this structural base with the target site of the referred macromolecules. Since this is where the catalytic mechanism of folate reduction occurs [36], it can be observed in folic acid (DHFR) and biopterin (PTR-1) agonists and in the reference molecule MTX (Figure 9).

### 3.2. Geometric Optimization of Structures

The Chem-Sketch software (ACD/Chemsketch Freeware, version 2018.1: Advanced Chemistry Development, Inc., Toronto, ON, Canada, 2019) was used for geometric design and optimization of selected molecules [37], which uses a 3D optimization algorithm, the force field based on parameterization CHARMM [38].

### 3.3. Building a Pharmacophoric Model

After the geometric optimization of the structures, in the molecular modeling program Discovery Studio Visualizer (version 19.1.0), the molecules were superimposed and saved in a single file in “.mol2” format for later input on the Pharmagist online server [25,39].

The Pharmagist online server calculated multiple flexible alignments of the selected ligands and ranked the best overlaps (score) by analyzing points in common with the MTX reference ligand, thus resulting in the pharmacophores and their structural characteristics [39] (Table 10). These structural characteristics were important to refine the search for compounds, on VS, to favor a pharmacokinetic profile within the parameters of Lipinski’s “Rule of 5” [40], which is a set of criteria used in the field of medicinal chemistry to assess the suitability of a molecule as a potential drug.

These rules were developed based on statistical analysis of a large set of successful pharmaceutical compounds. Although they are not an absolute guarantee that a molecule will be an effective drug [41], Lipinski’s rule is widely used as an initial tool to assess the viability of a molecule as a drug candidate.

### 3.4. Pharmacophore-Based Virtual Screening

A strategy adopted for further refinement in obtaining compounds with similar characteristics in the virtual screening was the construction of filters with minimum and maximum values of the physicochemical and structural properties of the molecules used in the pharmacophoric model. With the information obtained from the Pharmagist and using the Molinspiration online servers (https://molinspiration.com/ and Protox II (https://tox-new.charite.de/protox_II/, accessed on 9 February 2024), data were collected as follows: molecular weight, bond rotation, LogP (on Molinspiration), polar surface area, number of aromatic rings, hydrogen bond acceptor and hydrogen bond donor groups [42,43], thus generating a parameter for obtaining “Hits” with similar characteristics on the VS in the Pharmit molecule bank [44] (Table 11).

Gathering structural, physicochemical information and pharmacophoric groups, the search for “hits” that understood these requirements began in the Pharmit online molecule bank, precisely in the Molport company database (https://www.molport.com/).

### 3.5. Tanimoto Similarity

Two types of searches were carried out: Search 1, with a filter, using the parameter of values obtained from Molinspiration, and Search 2, without a filter. The hits obtained from the two searches were saved and on the Bindingdb web server they were submitted separately to Tanimoto Similarity studies, with the objective of obtaining hits with greater structural similarities to the reference molecule (MTX), in which similarity indexes that were greater than or equal to 0.6 (60% similarity) were selected, thus starting the second stage of the study [45,46].

### 3.6. Pharmacokinetic and Toxicological Predictions

The structures obtained on VS were prepared for pharmacokinetic and toxicological predictions. Using the PreADMET online server (https://preadmet.webservice.bmdrc.org/), the hits selected after similarity studies were first submitted to pharmacokinetic predictions, evaluating criteria such as plasma protein binding (PPB), blood–brain barrier penetration (BBB) [47], human intestinal absorption (HIA) [48], permeability of Caco2 cells, which predicts permeability in differentiated cells of the intestinal epithelium [49], and permeability of MDCK cells used as a model for permeability measurements in membranes.

In toxicological predictions, the Ames test was evaluated, which aims to predict whether a molecule or chemical product has mutagenic capacity for the genetic material of the tested organism, and the carcinogenic profile in rodents (mice and rats). It was also possible to obtain the toxicity class and the lethal dose (LD_50_) in mg/Kg via Protox ll online server [50].

### 3.7. Biological Activity and Cytotoxic Effect Predictions

PASSonline (http://way2drug.com/passonline/, accessed on 15 February 2024) is a website that allows for estimating the likely profile of biological activity of an organic compound (whose molecular mass is between 50 and 1250 Da) based on its structural formula [51]. It is possible to predict more than 4000 types of biological activity, including pharmacological effects, mechanisms of action, cytotoxic and adverse effects, interaction with metabolic enzymes and transporters, influence on gene expression, etc., with prediction of up to 95% accuracy [52].

A spectrum of biological activity is generated for the evaluated substance, and a list of types of biological activity for which it is likely to be active (Pa) and the probability of not being active (Pi) are calculated. In PASSonline, Pa and Pi values are independent, and their values vary from 0 to 1. By default, the value of Pa = Pi is defined as a threshold; therefore, all compounds with Pa > Pi are suggested as active [53].

Biological activity spectra were predicted for all promising compounds in PASSonline, where the structures that showed folate antagonist activity, DHFR inhibitor, PTR-1 inhibitor and antiprotozoal activity were selected for predictions of cytotoxic effect, which were performed via CLC-Pred (Cell Line Cytotoxic Preditor) (http://www.way2drug.com/cell-line/, accessed on 18 February 2024), which indicated the probability of cytotoxicity of chemical compounds in non-transformed or cancerous cell lines.

### 3.8. Molecular Docking

After biological activity predictions, the selected molecules were subjected to molecular docking assays. Obtaining the crystallographic structures of the molecular targets (available on the PDB website), it was possible to evaluate the interactions of the promising molecules with the active site of the enzymes selected for this study.

The docking tests were carried out using the Auto-Dock/Vina software in the PYRX version 0.8 graphical interface [54], using, as a target, the crystallographic structure of PTR-1 from *Leishmania major* complexed with MTX, PDB ID: 1E7W with resolution of 1.75 Å [55]; crystal structure of human DHFR complexed with MTX, PDB ID: 1DLS with resolution of 2.30 Å [56]; and the crystallographic structure of the enzyme human thymidylate synthase (TS) complexed with MTX, PDB ID: 5X66 with resolution of 1.99 Å [57]. AutoDock is a group of tool resources that allow the interaction between ligand and macromolecule and provides combinations with algorithm options: simulated annealing (SA), genetic algorithm (GA), and Lamarckian genetic algorithm (LGA) [54]. In this study, the Lamarckian genetic algorithm (LGA) was used, with standard parameters of the genetic algorithm (with a population size of 150), a maximum number of evaluations of 250,000, a maximum number of generations of 27,000, and a crossing rate of 0.8. The coordinates of the active sites used for each target are described in Table 12.

Ten molecular docking assays were conducted for each target compound, and their respective binding affinity results were presented as the arithmetic mean. The efficiency of this ligand/target coupling was validated by comparing the poses obtained by the Autodock software with the theoretical ones described in the literature. RMSD values (mean square deviation) were obtained and interpreted, being considered satisfactory when they presented values below 2Å [58].

### 3.9. Prediction of Lipophilicity and Water Solubility and Predicted Synthetic Accessibility (SA)

SwissADME (http://www.swissadme.ch/, accessed on 2 March 2024) was used to predict the lipophilicity and water solubility of compounds M601, M692, M700, and M703 expressed by logP and logS values, respectively, according to the methodological proposal of Ramos et al., 2022 [59]. It was also possible to use the same software to make predictions of synthetic accessibility, which gives a score between 1 (easy synthesis) and 10 (very difficult synthesis). This score is based on the fragmented analysis of the structures of more than 13 million compounds, with the hypothesis that the more frequent a molecular fragment is, the easier it is to obtain the molecule [33].

### 3.10. Structure–Activity Relationship (SAR) and Molecular Overlay of Promising Molecules

Data on the structure–activity relationship (SAR) were collected according to the methodology proposed by Ferreira et al. [15], whereby a structural search of the promising compounds was carried out in the Scifinder^®^ (https://scifinder.cas.org/, accessed on 28 March 2024) database to check if there is additional information and experiments with biological activities in the literature on the promising structures [60].

The molecular overlay was carried out following the methodological proposal of Lima et al. [61]. The molecules resulting from the virtual screening were superimposed with two or more three-dimensional chemical structures, considering the contributions (%) of the steric and electronic fields, to evaluate similarities with the pivotal structure. The analyses were performed using Biovia Discovery Studio Visualizer (Dassault Systémes, version 19.1.0, Vélizy-Villacoublay, France) [62] software, considering the contributions of 25%, 50%, 75% and 100% of the electronic field.

## 4. Conclusions

This study successfully identified promising PTR1 inhibitors for *Leishmania major* through an integrated in silico approach combining virtual screening, pharmacokinetic and toxicological predictions, and molecular docking analyses. The compounds M601, M692, M700, and M703 exhibited strong binding affinities and significant interactions with key residues of the target enzymes, with M692 standing out for its potential multitarget profile. 

In addition to favorable binding properties, the ADMET predictions revealed good intestinal absorption, moderate plasma protein binding, low blood–brain barrier penetration, and a markedly improved toxicological profile compared to methotrexate. These findings highlight the robustness of the computational pipeline employed, which enabled the rational selection of candidates with high therapeutic potential and low toxicological risk, which allows for expanding the applicability of the method of this study to other therapeutic targets such as trypanothione reductase, responsible for protection against oxidative stress, and topoisomer l, which has differences with its mammalian counterparts. However, there are limitations that need to be evaluated, such as the simplified biological complexity in in silico models that can lead to inaccurate results. Overall, this work provides a solid foundation for advancing these molecules into experimental validation, contributing to the development of safer and more effective therapeutic alternatives for the treatment of leishmaniasis.

## Figures and Tables

**Table 1:** Best alignment score of selected compounds generated by Pharmagist.

Score	F	SF	Aro	Hyd	Don	Acc	Neg	Pos	Aligned Compounds
72,746	11	11	3	0	2	6	0	0	1 *, 2, 3, 5, 6, 7, 8, 9, 10

Abbreviations: F: Total features; SF: Total spatial features; Aro: Aromatic groups; Hyd: Hydrophobic groups; Don: Hydrogen bond donor groups; Acc: Hydrogen bond acceptor groups; Neg: Anionic atoms; Pos: Cationic atoms; * Reference structure.

**Table 2:** Physicochemical properties of reference structures.

Structures	MolWeight	RotBonds	LogP	PSA	Aromatics	HBA	HBD
MTX	454.45	9	−1.97	210.55	3	9	3
1	480.53	8	0.25	173.69	3	8	3
2	450.50	6	0.88	153.47	3	7	2
3	464.53	7	1.48	162.25	3	7	3
4	464.53	7	1.48	162.25	3	7	3
5	436.48	6	0.63	162.25	3	7	3
6	522.57	10	1.12	179.77	3	9	2
7	437.46	6	0.10	175.15	3	8	3
8	421.46	5	−0.50	179.04	3	6	4
9	422.45	5	0.02	173.25	3	7	3
Minimum	421.46	5	−1.97	153.47	3	7	2
Maximum	522.57	10	1.48	210.55	3	9	4

Abbreviations: MTX: Methotrexate; MolWeight: Molecular weight; RotBond: bond rotation; PSA: Polar surface area; Aromatics: Aromatic rings; HBA: Hydrogen bond acceptor; HBD: Hydrogen bond donor.

**Table 3:** Absorption and distribution properties of selected compounds.

Hits	Absorption			Distribution	
	HIA (%)	CaCo-2 (nm/s)	MDCK (nm/s)	PPB (%)	BBB (%)
MTX (Pivot)	36.61	18.89	2.45	44.50	0.04
MGL	2.72	20.12	0.52	96.57	0.04
ANF B	4.70	14.14	0.046	39.01	0.03
PTM	86.27	21.08	0.52	3.17	0.03
BDB-1	83.23	18.27	1.64	48.12	0.05
MolPort-001-684-165	26.02	16.52	6.59	45.73	0.07
MolPort-001-684-168	23.34	17.00	4.84	48.51	0.05
MolPort-001-684-171	22.81	17.30	4.79	40.77	0.04
MolPort-004-285-551	23.37	17.00	4.84	48.51	0.05
MolPort-004-955-331	82.2	19.87	5.34	61.46	0.06
MolPort-004-964-256	23.47	17.30	4.79	40.81	0.04
MolPort-005-942-787	65.57	21.08	0.57	82.48	0.06
MolPort-005-942-788	88.36	19.51	0.08	86.03	0.09
MolPort-006-169-571	71.67	18.46	4.47	63.84	0.05
MolPort-008-010-692	94.88	24.51	212.65	88.49	0.09
MolPort-008-010-700	94.98	34.78	11.48	86.68	0.11
MolPort-008-010-703	94.92	28.48	79.41	87.64	0.12
MolPort-009-679-397	50.35	19.61	0.07	77.43	0.04
MolPort-035-395-047	50.35	19.61	0.07	77.43	0.04
MolPort-035-776-124	71.93	19.02	23.68	46.05	0.05
MolPort-046-592-422	88.42	19.59	0.14	84.94	0.07
MolPort-046-684-424	81.26	20.19	0.06	79.26	0.05
MolPort-047-399-601	79.50	20.20	0.37	67.52	0.04

Abbreviations: HIA: Human intestinal absorption; CaCo-2: Cancer coli, “colon cancer cell”; MDCK: Madin-Darby canine kidney; PPB: Plasma protein binding; BBB: blood–brain barrier penetration; MTX: Methotrexate; MGL: Meglumine antimoniate; ANF B: Amphotericin B; PTM; Pentamidine; BDB-1: The most active molecule from the reference structures group, BDBM50398391.

**Table 4:** Properties of chemical structures obtained from toxicological predictions.

Structures	Carcinogenicity ^a^		Ames Test ^a^	LD_50_ (mg/Kg) ^b^	Toxicity Class ^b^
	Mouse	Rat	Mutagenicity		
MTX	Negative	Negative	Mutagenic	3	1
MGL	Out of range	Out of range	Mutagenic	1.500	4
ANF B	Positive	Positive	Mutagenic	100	3
PTM	Positive	Negative	Mutagenic	1.086	4
BDB-1	Negative	Negative	Mutagenic	135	3
MolPort-008-010-692 *	Positive	Negative	Mutagenic	135	3
MolPort-008-010-700 *	Positive	Negative	Non-mutagenic	3.000	5
MolPort-008-010-703 *	Positive	Negative	Non-mutagenic	3.000	5
MolPort-047-399-601 *	Negative	Positive	Mutagenic	135	3

* Selected hits; ^a^ PreADMET; ^b^ Protox-II.

**Table 5:** Results of biological activity predictions obtained in PASSonline.

Chemical Structure	Biological Activity	Pa ^a^	Pi ^b^
MTX	Dihydrofolate reductase inhibitor	0.913	0.001
	Folate antagonist	0.881	0.002
	Thymidylate synthase inhibitor	0.549	0.001
	Pteridine reductase inhibitor	0.507	0.003
	Antiprotozoal (Toxoplasma)	0.452	0.007
	Antiprotozoan (Trypanosoma)	0.364	0.054
M601	Folate antagonist	0.385	0.006
	Pteridine reductase inhibitor	0.264	0.009
M692	Folate antagonist	0.396	0.006
	Pteridine reductase inhibitor	0.360	0.005
	Antiprotozoan (Trypanosoma)	0.376	0.048
	Antiprotozoal (Toxoplasma)	0.288	0.019
	Antiprotozoal (Plasmodium)	0.228	0.036
M700	Antiprotozoan (Trypanosoma)	0.430	0.031
	folate antagonist	0.374	0.007
	Pteridine reductase inhibitor	0.218	0.013
M703	Antiprotozoan (Trypanosoma)	0.531	0.015
	folate antagonist	0.331	0.010
	Antiprotozoal (Toxoplasma)	0.331	0.013
	Antiprotozoal (Plasmodium)	0.295	0.015
	Pteridine reductase inhibitor	0.236	0.011

^a^ Pa = Probability to be active; ^b^ Pi = Probability to be inactive.

**Table 6:** Results of cytotoxic effect predictions of promising structures.

Chemical Structure	Tissues	Pa ^a^	Pi ^b^	Cell Line	Tumor Type
MTX	Brain	0.856	0.001	D54	Glioblastoma
	Hematopoietic and lymphoid tissue	0.739	0.000	Manca	Lymphoma
	Blood	0.662	0.006	CCRF-CEM	Leukemia
	Blood	0.609	0.014	MOLT-4	Leukemia
M601	Brain	0.317	0.007	D54	Glioblastoma
	Pancreas	0.322	0.122	CFPAC-1	Carcinoma
	Skin	0.334	0.217	SK-MEL-1	Melanoma
	Skin	0.285	0.176	A2058	Melanoma
	Hematopoietic and lymphoid tissue	0.153	0.049	U-937	Lymphoma
M692	Bone	0.549	0.004	HOS	Sarcoma
	Kidney	0.549	0.015	786-0	Carcinoma
	Colon	0.511	0.026	HCT-116	Carcinoma
M700	Lung	0.532	0.026	DMS-114	Carcinoma
	Bone	0.509	0.005	HOS	Sarcoma
	Colon	0.502	0.027	HCT-116	Carcinoma
M703	Lung	0.536	0.004	HOS	Sarcoma
	Bone	0.525	0.029	DMS-114	Carcinoma
	Colon	0.509	0.026	HCT_116	Carcinoma

^a^ Pa = Probability to be active; ^b^ Pi = Probability to be inactive.

**Table 7:** Lipophilicity of promising compounds.

Structure	iLOG	XLOGP	WLOGP	MLOGP	SILICOS-IT	Consensus LogP
MTX (Pivot)	0.22	−2.62	−0.52	−1.52	−1.01	−1.09
BDB-1	2.18	−0.10	0.09	0.20	0.21	0.52
M601	2.99	0.96	0.50	−1.41	1.52	0.91
M692	2.59	2.41	2.35	0.94	2.46	2.15
M700	3.48	3.45	3.45	1.41	2.73	2.90
M703	3.11	3.48	3.44	1.71	2.68	2.88

iLOGP: physics-based method relying on free energies of solvation in n-octanol and water calculated by the generalized-born and solvent-accessible surface area model; XLOGP: an atomistic method including corrective factors and knowledge-based library; WLOGP: implementation of a purely atomistic method based on the fragmental system of Wildman and Crippen; MLOGP: an archetype of a topological method relying on a linear relationship with 13 molecular descriptors; SILICOS-IT: a hybrid method relying on 27 fragments and 7 topological descriptors.

**Table 8:** Water solubility and prediction of synthetic accessibility (SA) of promising compounds.

Structure	ESOL	Ali	SILICOS-IT	Consensus LogS ^1^	SA ^2^ Score
MTX (Pivot)	−1.03 (s)	−2.65 (s)	−4.72 (ms)	−2.80 (s)	4.49
BDB-1	−2.50 (s)	−3.09 (s)	−4.72 (ms)	−3.43 (s)	3.42
M601	−4.33 (ms)	−5.78 (ms)	−7.26 (ms)	−5.79 (ms)	4.75
M692	−3.72 (ms)	−3.95 (ms)	−8.12 (ps)	−5.26 (ms)	2.84
M700	−4.72 (ms)	−5.11 (ms)	−8.31 (ms)	−6.05 (ps)	3.09
M703	−4.46 (ms)	−4.94 (ms)	−8.21 (ps)	−5.87 (ms)	2.85

^1^ Solubility (s = soluble, ms = moderately soluble, ps = poorly soluble); ^2^ Synthetic accessibility (SA): SwissADME—SA scores range from 1 (very easy) to 10 (very difficult).

**Table 9:** Steric and electrostatic molecular field overlaps analyzed by Discovery Studio for the pivot structure (MTX), the most active molecule of the studied series (BDB-1), and the promising compounds identified in the MolPort^®^ database.

Structure		Overlay			
	Molecules	25% ^a^	50% ^b^	75% ^c^	100% ^d^
MTX	M601	0.58	0.50	0.42	0.35
	M692	0.43	0.40	0.36	0.37
	M700	0.51	0.44	0.40	0.37
	M703	0.52	0.45	0.39	0.34
BDB-1 *	M601	0.51	0.48	0.44	0.41
	M692	0.46	0.41	0.36	0.32
	M700	0.51	0.43	0.37	0.35
	M703	0.56	0.49	0.39	0.40

^a^ 75%/25% steric and electrostatic contribution, respectively; ^b^ 50%/50% electrostatic contribution; ^c^ 25%/75% electrostatic contribution; ^d^ 100% electrostatic contribution; * Most active molecule of the studied series.

**Table 10:** Structural and pharmacophoric characteristics obtained in Pharmagist, showing, from left to right, total number of atoms (Atm), total number of spatial features (SF), number of aromatic rings (Aro), hydrophobic groups (Hyd), hydrogen bond donor group (Don), hydrogen bond acceptor group (Acc), and negatives (Neg) and positives (Pos).

Molecules	Atm	SF	Aro	Hyd	Don	Acc	Neg	Pos
MTX (pivot)	53	18	3	1	3	9	2	0
01	63	14	3	1	3	8	0	0
02	59	14	3	2	2	7	0	0
03	62	17	3	4	3	7	0	0
04	62	17	3	4	3	7	0	0
05	56	14	3	1	3	7	0	0
06	68	16	3	2	2	9	0	0
07	55	15	3	1	3	8	0	0
08	54	13	3	0	4	6	0	0

**Table 11:** Maximum and minimum values of the physicochemical and structural properties of the molecules used in the generation of the pharmacophoric model.

Min		Max
421.46	Molweight	522.57
5.00	Rotbonds	10.00
−1.97	LogP	1.48
153.47	PSA	210.55
3.00	Aromatics	3.00
7.00	HBA	9.00
2.00	HBD	4.00

**Table 12:** Coordinates of active sites of PTR-1, DHFR, and TS enzymes.

Target	Ligand	Grid Center (Å)	Grid Size (Å)
Pteridine reductase-1		X: −6.4371	42
PDB ID: 1E7W	MTX	Y: 21.1583	29
Resolution: 1.75 Å		Z: 26.5948	42
Dihydrofolate reductase		X: 29.9128	42
PDB ID: 1DLS	MTX	Y: 17.3729	30
Resolution: 2.30 Å		Z: −2.4309	42
Thymidylate synthase		X: 155.937	48
PDB ID: 5X66	MTX	Y: 150.630	23
Resolution: 1.99 Å		Z: 24.6042	23

## Data Availability

Data can be found in this article and the Supplementary Materials.

## Supplementary Materials

The following supporting information can be downloaded at: https://www.mdpi.com/article/10.3390/ph18081237/s1. Table S1: Compounds selected from the BindingDB database with their respective inhibition constant (Ki) values; Table S2: Interactions between amino acid residues of the PDB 1DLS active site with MTX; Figure S1: 3D Interactions (1DLS-MTX)/2D Interactions (1DLS-MTX); Table S3: Interactions between amino acid residues of the PDB 1DLS active site with M601: Figure S2: 3D Interactions (1DLS-M601)/2D Interactions (1DLS-M601); Table S4: Interactions between amino acid residues of the PDB 1DLS active site with M692; Figure S3: 3D Interactions (1DLS-M692)/2D Interactions (1DLS-M692); Table S5: Interactions between amino acid residues of the PDB 1DLS active site with M700; Figure S4: 3D Interactions (1DLS-M700)/2D Interactions (1DLS-M700); Table S6: Interactions between amino acid residues of the PDB 1DLS active site with M703; Figure S5: 3D Interactions (1DLS-M703)/2D Interactions (1DLS-M703); Table S7: Interactions between amino acid residues of the PDB 1E7W active site with MTX; Figure S6: 3D Interactions (1E7W-MTX)/2D Interactions (1E7W-MTX); Table S8: Interactions between amino acid residues of the PDB 1E7W active site with M601; Figure S7: 3D Interactions (1E7W-M601)/2D Interactions (1E7W-M601); Table S9: Interactions between amino acid residues of the PDB 1E7W active site with M692; Figure S8: 3D Interactions (1E7W-M692)/2D Interactions (1E7W-M692); Table S10: Interactions between amino acid residues of the PDB 1E7W active site with M700; Figure S9: 3D Interactions (1E7W-M700)/2D Interactions (1E7W-M700); Table S11: Interactions between amino acid residues of the PDB 1E7W active site with M703; Figure S10: 3D Interactions (1E7W-M703)/2D Interactions (1E7W-M703); Table S12: Interactions between amino acid residues of the PDB 5X66 active site with MTX; Figure S11: 3D Interactions (5X66-MTX)/2D Interactions (5X66-MTX); Table S13: Interactions between amino acid residues of the PDB 5X66 active site with M601; Figure S12: 3D Interactions (5X66-M601)/2D Interactions (5X66-M601); Table S14: Interactions between amino acid residues of the PDB 5X66 active site with M692; Figure S13: 3D Interactions (5X66-M692)/2D Interactions (5X66-M692); Table S15: Interactions between amino acid residues of the PDB 5X66 active site with M700; Figure S14: 3D Interactions (5X66-M700)/2D Interactions (5X66-M700); Table S16: Interactions between amino acid residues of the PDB 5X66 active site with M703; Figure S15: 3D Interactions (5X66-M703)/2D Interactions (5X66-M703).
