# Supplementary material for: Identification of Potential Pteridin Reductase-1 Inhibitors for the Treatment of Leishmaniasis: A Bioinformatics Approach
Source: Pharmaceuticals (Basel). 2025 Aug 21;18(8):1237. doi: 10.3390/ph18081237 (PMC12389166; doi:10.3390/ph18081237)
Supplement: Supplementary file 1 [file pharmaceuticals-18-01237-s001.zip › pharmaceuticals-3705418-supplementary.pdf]

Supplementary Materials

Identification of potential Pteridin Reductase-1 inhibitors for the treatment of leishmaniasis: a bioinformatics approach

Paulo R. da S. R. Júnior[1,2], Lúcio R. de Lima[2], Luciane B. Silva[1,2], Jorddy N. da Cruz[2], Njogu M. Kimani[3], Gustavo H. G. Trossini[4], Cleison C. Lobato[2], Cleydson Breno R. dos Santos[1,2*].

[1] Graduate Program in Pharmaceutical Sciences, Federal University of Amapá, Macapá, 68902-280, AP, Brazil. e-mail@e-mail.com
[2] Laboratory of Modeling and Computational Chemistry, Department of Biological and Health Sciences, Federal University of Amapá, Macapá 68902-280, AP, Brazil.
[3] Natural Product Chemistry and Computational Drug Discovery Laboratory, Department of Physical Sciences, University of Embu, P.O. Box 6-60100, Embu, Kenya.
[4] Laboratory of Integration Techniques Experimental and Computational in Drug Design, Department of Pharmacy, School of Pharmaceutical Sciences, University of São Paulo, Av. Prof. Lineu Prestes 580, 05508-900 São Paulo-SP, Brazil.
* Correspondence: breno@unifap.br

Table S1: Compounds selected from the BindingDB database with their respective inhibition constant (Ki) values.

Nº	BindingDB identification code	Structures	Ki (nM)
01	BDBM50398391	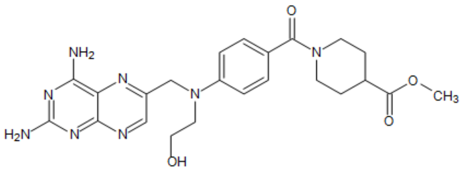	30
02	BDBM50398394	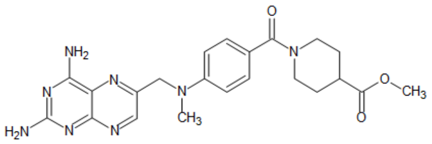	37
03	BDBM50398392	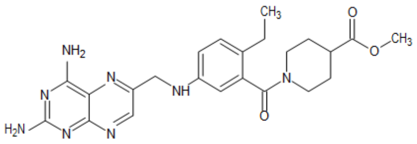	60
04	BDBM50398390	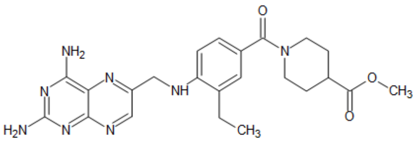	78
05	BDBM50398395	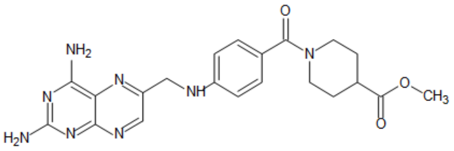	100
06	BDBM50398389	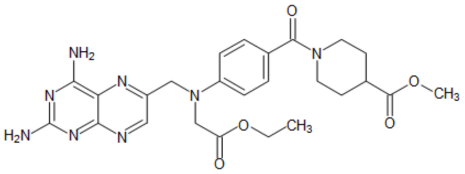	100

07	BDBM50398396	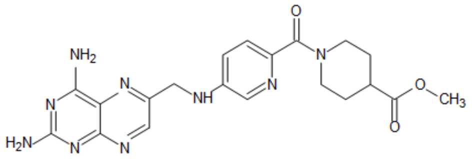	210
08	BDBM50398393	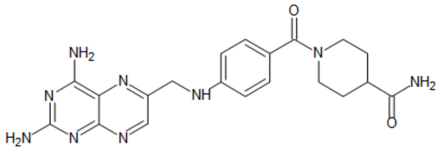	390
09	BDBM50398388	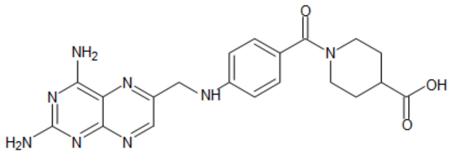	4.17x10³

Molecular Docking Results

Interactions between the amino acid residues of PDBs (1DLS, 1E7W and 5X66) with Methotrexate (MTX) and the proposed substances (M601, M692, M700 and M703).

PDB: 1DLS
Ligand: MTX
Binding affinity: -10,4 Kcal/mol

Table S2: Interactions between amino acid residues of the PDB 1DLS active site with MTX.

Residues	Interactions	Bond length (Å)
ILE7	Pi-Alkyl Conventional<br>Hydrogen Bond	5,14<br>2,89
ALA9	Pi-Alkyl	4,26/5,18
ARG28	Conventional Hydrogen Bond	3,13
GLU30	Conventional Hydrogen<br>Bond Unfavorable Acceptor-Acceptor	2,73<br>2,69
PHE31	Pi-Donor Hydrogen Bond	4,12
SER59	Carbon Hydrogen Bond	3,11
ILE60	Pi-Sigma	3,98
ASN64	Conventional Hydrogen Bond	2,64
ARG70	Conventional Hydrogen<br>BondSalt Bridge	3,12<br>2,68
VAL115	Conventional Hydrogen Bond	3,19

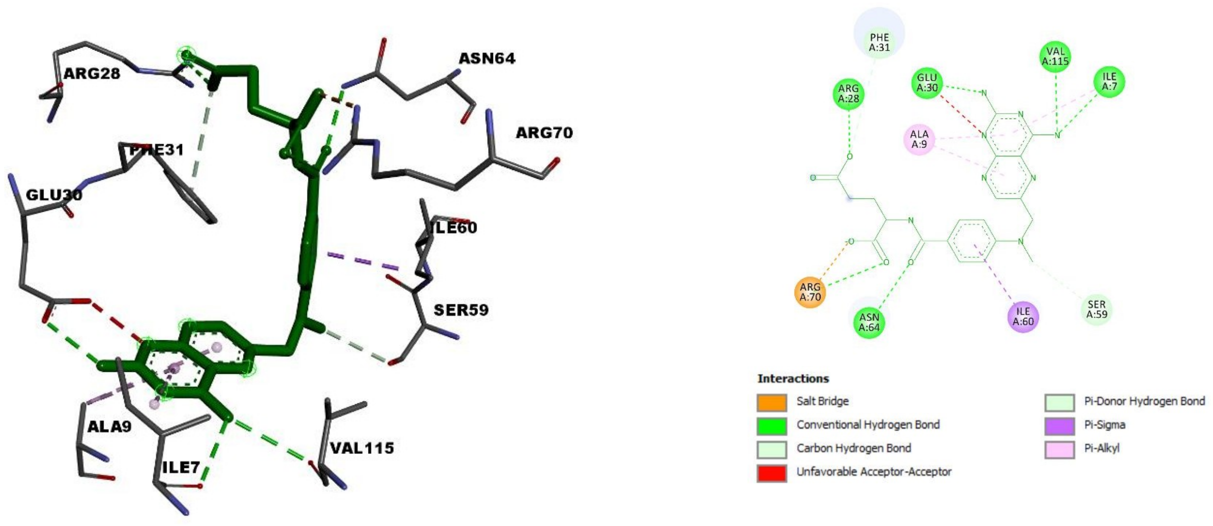

Figure S1: 3D Interactions (1DLS-MTX) 2D Interactions (1DLS-MTX)

PDB: 1DLS
Ligand: MolPort-047-399-601 (M601)
Binding affinity: -9,6 Kcal/mol

Table S3: Interactions between amino acid residues of the PDB 1DLS active site with M601.

Residues	Interactions	Bond length (Å)
ILE7	Pi-Alkyl	5,03
VAL8	Conventional Hydrogen Bond	3,06
ALA9	Pi-Alkyl	4,41/5,29
ARG28	Pi-Cation	3,66
GLU30	Conventional Hydrogen Bond	1,89/1,90
ILE60	Pi-Alkyl	4,04
ASN64	Conventional Hydrogen Bond	3,25/3,28
LYS68	Carbon Hydrogen Bond	3,49
THR136	Conventional Hydrogen Bond	2,92

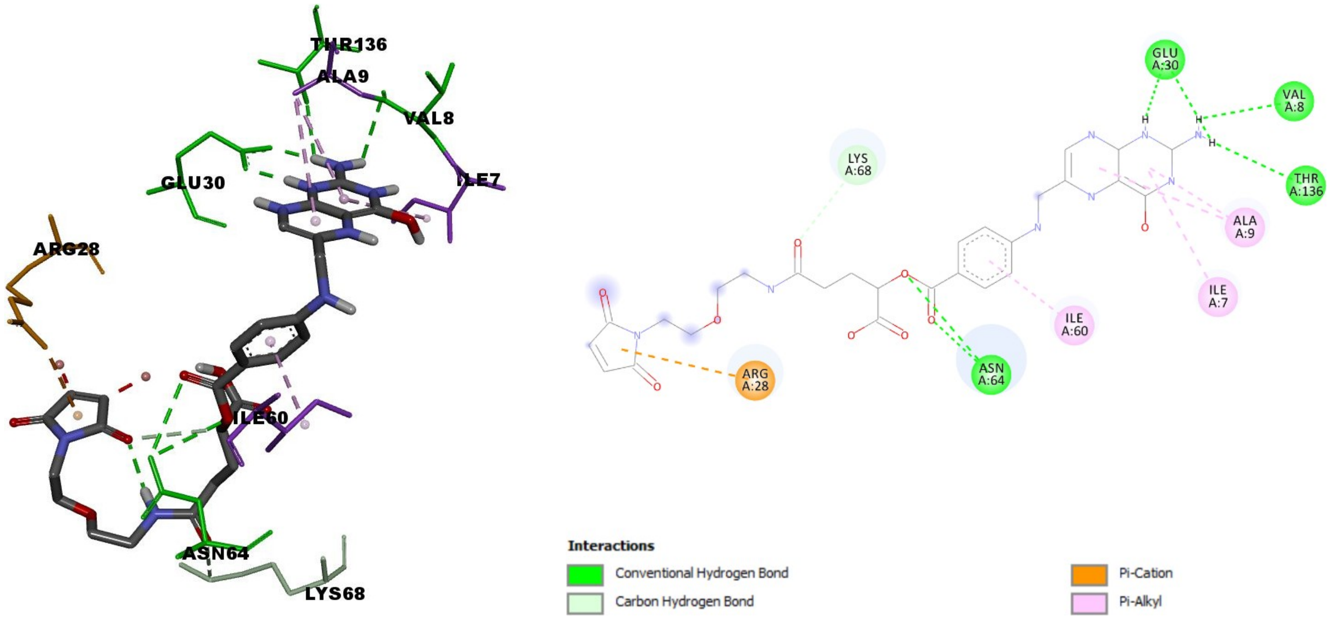

Figure S2: 3D Interactions (1DLS-M601) 2D Interactions (1DLS-M601)

PDB: 1DLS
Ligand: MolPort-008-010-692 (M692)
Binding affinity: -9,2 Kcal/mol

Table S4: Interactions between amino acid residues of the PDB 1DLS active site with M692.

Residues	Interactions	Bond length (Å)
TYR22	Conventional Hydrogen Bond	2,82
PHE31	Pi-Pi-Stacked	4,23/4,80
PHE34	Pi-Alkyl	4,99
THR56	Carbon Hydrogen Bond	3,71
SER59	Conventional Hydrogen Bond	2,82
ILE60	Pi-Sigma	3,95
PRO61	Pi-Alkyl	3,93/4,54
VAL115	Alkyl	5,35

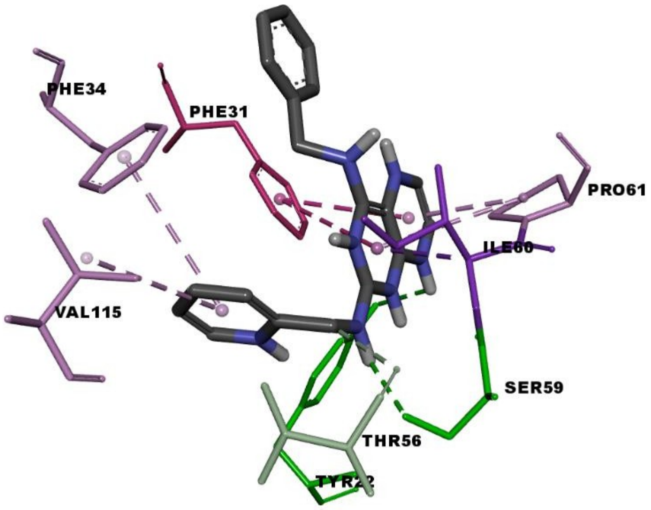

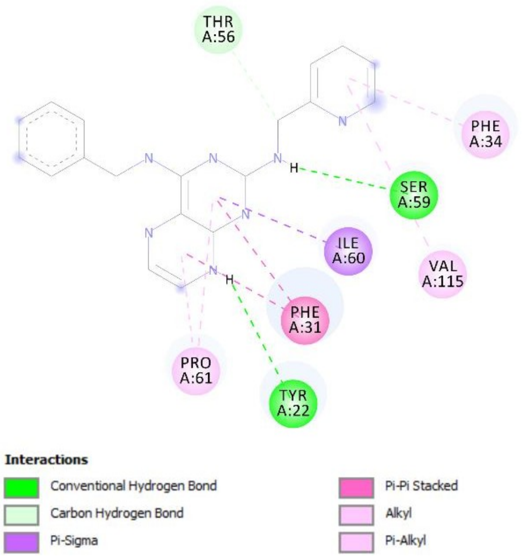

Figure S3: 3D Interactions (1DLS-M692) 2D Interactions (1DLS-M692)

PDB: 1DLS
Ligand: MolPort-008-010-700 (M700)
Binding affinity: -8,8 Kcal/mol

Table S5: Interactions between amino acid residues of the PDB 1DLS active site with M700.

Residues	Interactions	Bond length (Å)
ASP21	Conventional Hydrogen Bond	2,43
TYR22	Pi-Donor Hydrogen Bond	3,53
PHE31	Pi-Pi-Stacked	4,27/5,49
SER59	Conventional Hydrogen Bond<br>Carbon Hydrogen Bond	2,57<br>3,74
ILE60	Pi-Alkyl	4,50/4,58
PRO61	Pi-Alkyl	3,99

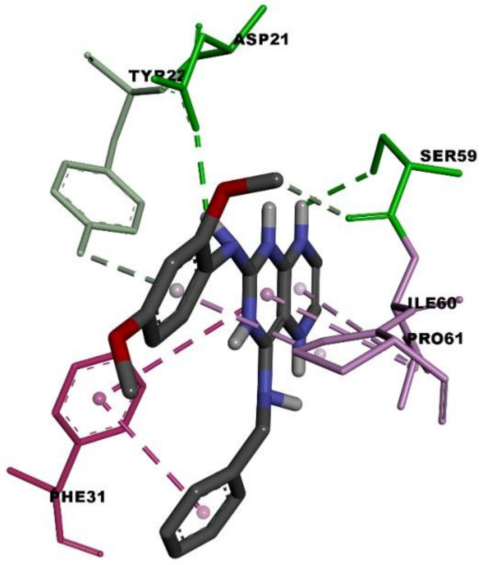

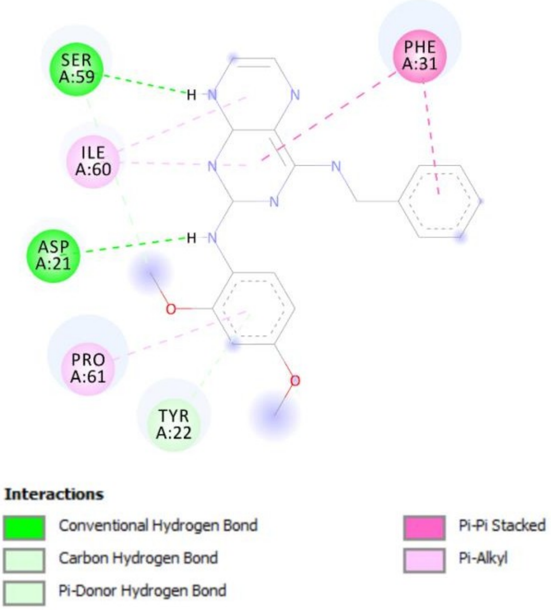

Figure S4: 3D Interactions (1DLS-M700) 2D Interactions (1DLS-M700)

PDB: 1DLS
Ligand: MolPort-008-010-703 (M703)
Binding affinity: -9,2 Kcal/mol

Table S6: Interactions between amino acid residues of the PDB 1DLS active site with M703.

Residues	Interactions	Bond length (Å)
TYR22	Pi-Donor Hydrogen Bond	4,00
PHE34	Pi-Pi-T-Shaped	5,28
SER59	Conventional Hydrogen Bond	2,66/2,67
ILE60	Pi-Alkyl	4,26
PRO61	Pi-Alkyl	4,14/5,01
LEU67	Pi-Alkyl	5,40

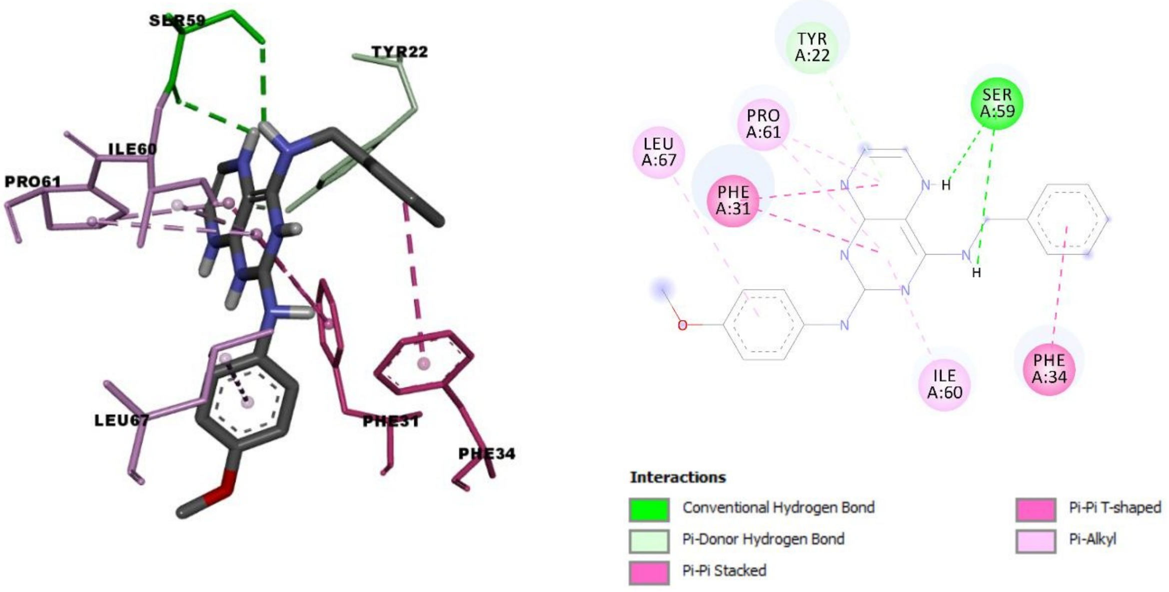

Figure S5: 3D Interactions (1DLS-M703) 2D Interactions (1DLS-M703)

PDB: 1E7W
Ligand: MTX
Binding affinity: -6,6 Kcal/mol

Table S7: Interactions between amino acid residues of the PDB 1E7W active site with MTX

Residues	Interactions	Bond length (Å)
SER111	Conventional Hydrogen Bond	2,64/2,85
PHE113	Pi-Pi Stacked	3,94/4,10
LEU188	Pi-Alkyl	5,13
TYR194	Conventional Hydrogen Bond	2,85
TYR191	Unfavorable Bump	2,07
TRP238	Pi-Pi Shaped	5,18

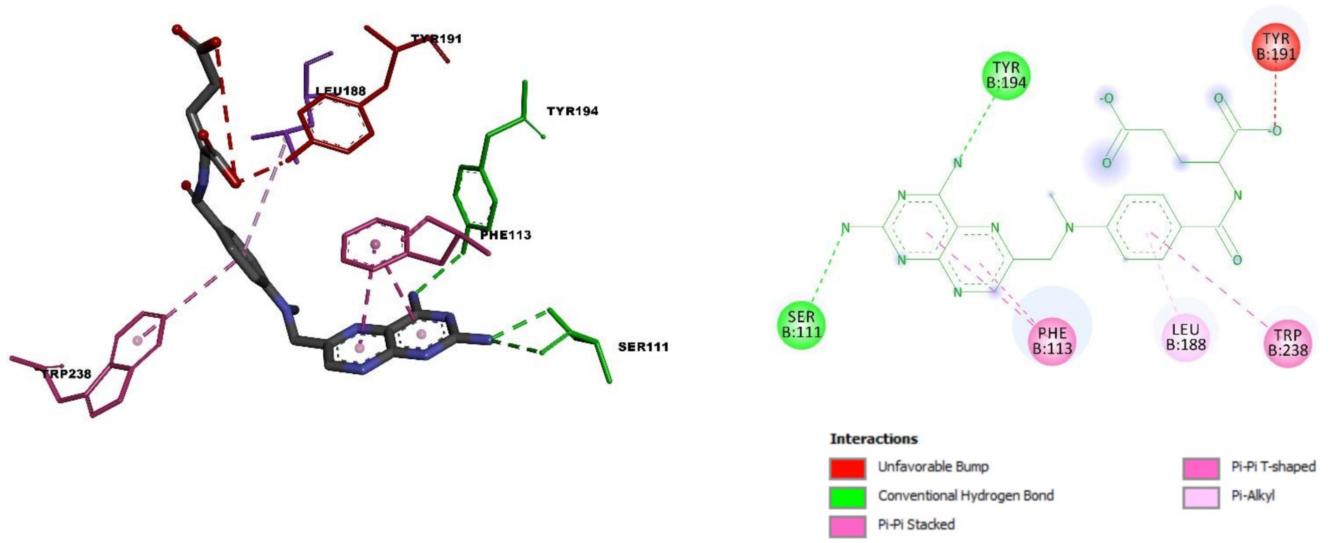

Figure S6: 3D Interactions (1E7W-MTX) 2D Interactions (1E7W-MTX)

PDB: 1E7W
Ligand: MolPort-047-399-601 (M601)
Binding affinity: -6,6 Kcal/mol

Table S8: Interactions between amino acid residues of the PDB 1E7W active site with M601.

Residues	Interactions	Bond length (Å)
ASN109	Conventional Hydrogen Bond	2,75
SER111	Conventional Hydrogen Bond	2,39
TYR191	Conventional Hydrogen Bond	2,44
TYR194	Conventional Hydrogen Bond	3,10
PRO224	Pi-Alkyl	4,72
GLY225	Carbon	3,40
SER227	Conventional Hydrogen Bond	2,20
LEU229	Pi-Sigma	3,96
TRP238	Pi-Stacked<br>Pi-Shaped	5,12<br>5,98

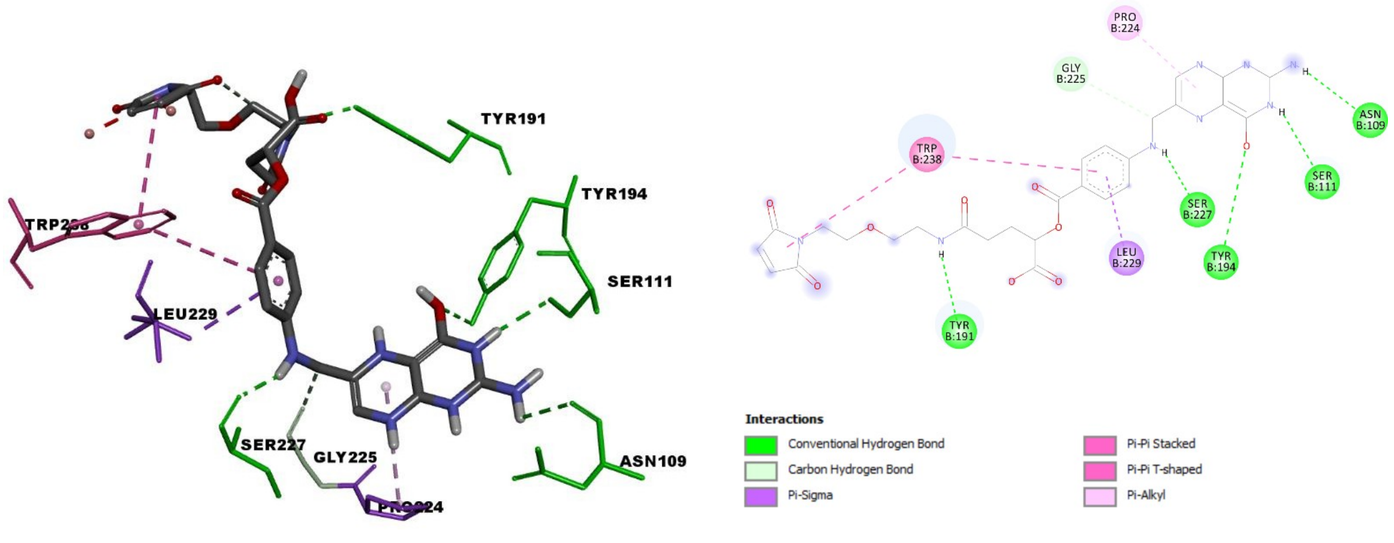

Figure S7: 3D Interactions (1E7W-M601) 2D Interactions (1E7W-M601)

PDB: 1E7W
Ligand: MolPort-008-010-692 (M692)
Binding affinity: -8,6 Kcal/mol

Table S9: Interactions between amino acid residues of the PDB 1E7W active site with M692.

Residues	Interactions	Bond length (Å)
PHE113	Pi-Pi-Shaped	5,36
ASP181	Pi-Anion	4,16/4,75
LEU188	Pi-Alkyl	5,48
PRO224	Alkyl	4,70
GLY225	Conventional Hydrogen Bond	1,90/2,64
LEU229	Pi-Alkyl	5,18

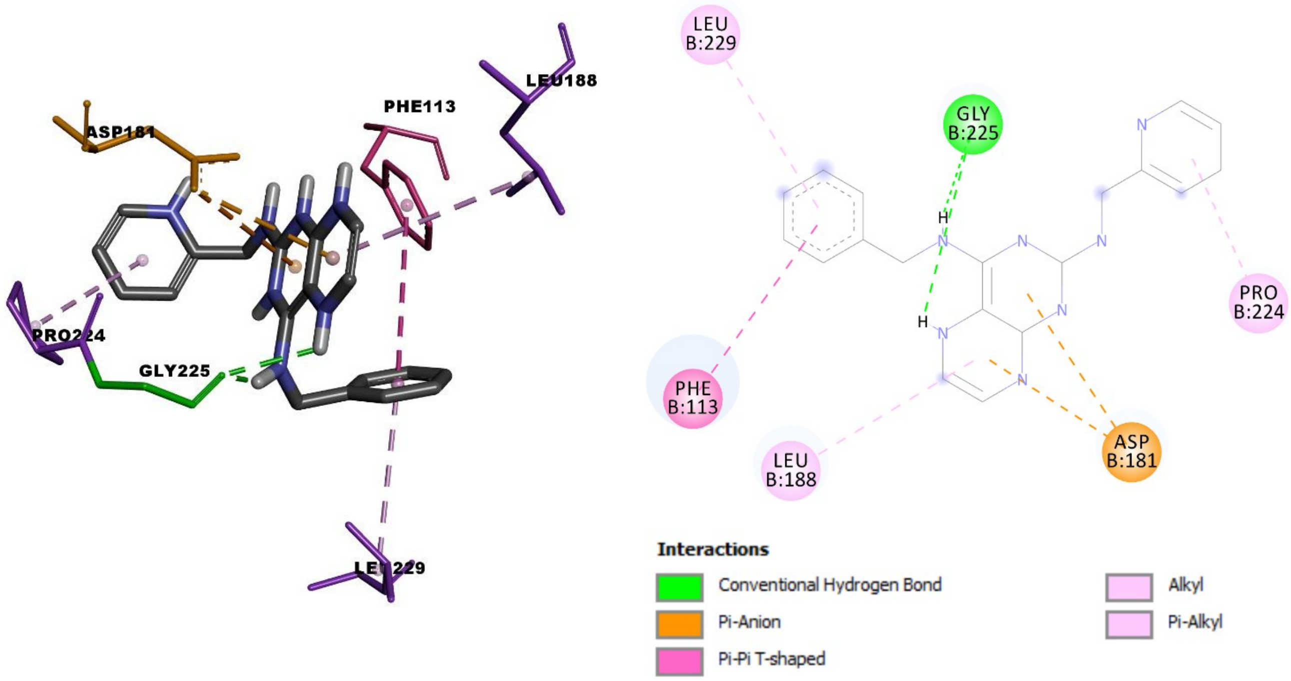

Figure S8: 3D Interactions (1E7W-M692) 2D Interactions (1E7W-M692)

PDB: 1E7W
Ligand: MolPort-008-010-700 (M700)
Binding affinity: -8,4 Kcal/mol

Table S10: Interactions between amino acid residues of the PDB 1E7W active site with M700.

Residues	Interactions	Bond length (Å)
PHE113	Pi-Pi-T-Shaped<br>Pi-Stacked	4,94<br>5,0
ASP181	Conventional Hydrogen Bond	2,68/1,91
LEU188	Pi-Alkyl<br>Pi-Sigma	5,39<br>3,93
TYR194	Conventional Hydrogen Bond	2,15
GLY225	Carbon Hydrogen Bond	3,53
LEU229	Pi-Alkyl	5,39
TRP238	Pi-Pi-T-Shaped	4,77

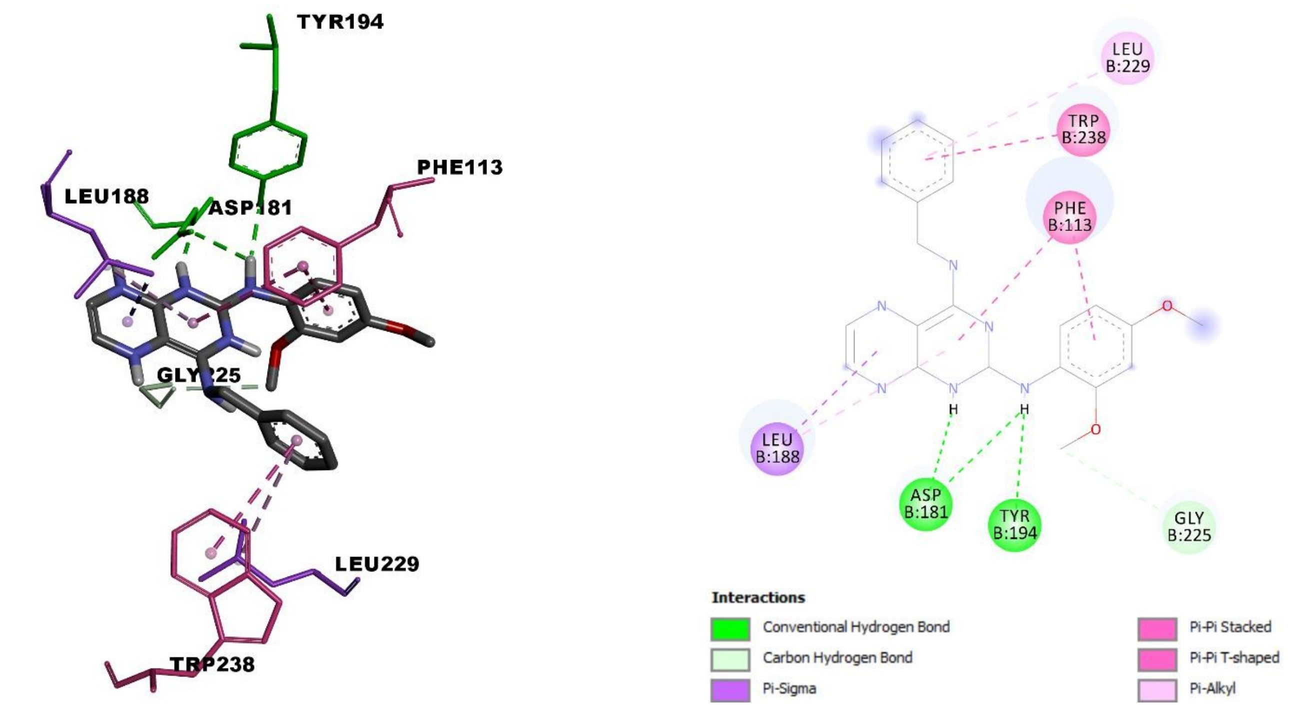

Figure S9: 3D Interactions (1E7W-M700) 2D Interactions (1E7W-M700)

PDB: 1E7W
Ligand: MolPort-008-010-703 (M703)
Binding affinity: -8,0 Kcal/mol

Table S11: Interactions between amino acid residues of the PDB 1E7W active site with M703.

Residues	Interactions	Bond length (Å)
SER111	Conventional Hydrogen Bond	3,33
PHE113	Pi-Pi-T-Shaped<br>Pi-Pi-Stacked	5,12<br>4,80/5,99
ASP181	Conventional Hydrogen Bond<br>Pi-Anion	1,97/2,75<br>4,99
LEU188	Pi-Sigma	3,98/3,98
TYR194	Pi-Donor Hydrogen Bond	2,15

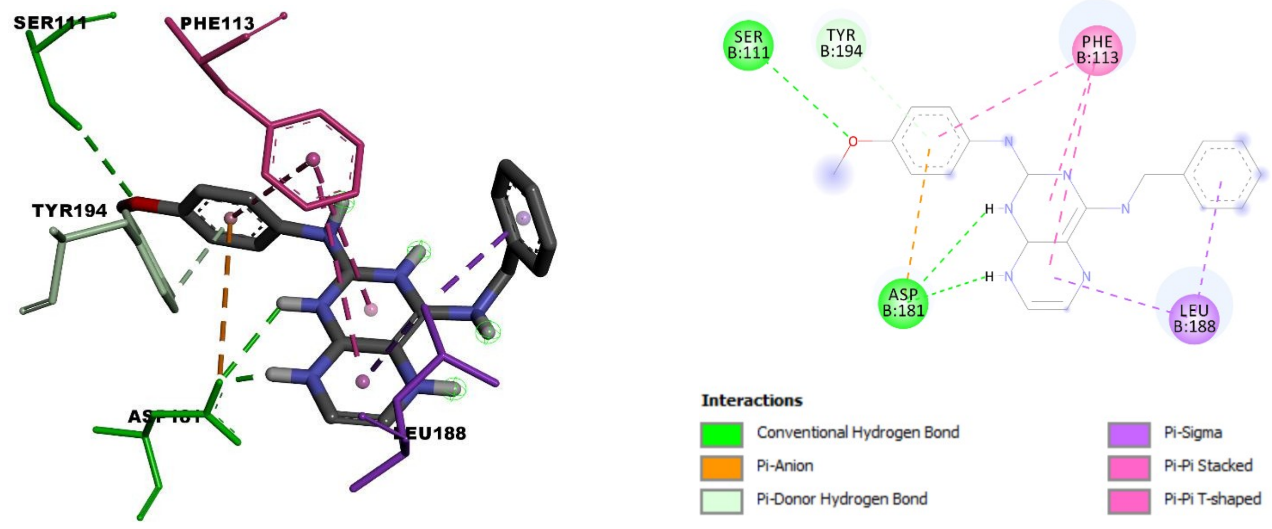

Figure S10: 3D Interactions (1E7W-M703) 2D Interactions (1E7W-M703)

PDB: 5X66
Ligand: MTX
Binding affinity: -8,1 Kcal/mol

Table S12: Interactions between amino acid residues of the PDB 5X66 active site with MTX.

Residues	Interactions	Bond length (Å)
ARG78	Attractive Charge Conventional<br>Hydrogen Bond	4,10<br>2,61
ASP218	Unfavorable Acceptor-Acceptor	2,70
ARG28	Conventional Hydrogen Bond	3,13
ILE108	Pi-Sigma	3,79
MET311	Pi-Sulfur<br>Pi-Alkyl	3,60<br>4,87
PHE225	Pi-Pi T-Shaped<br>Pi-Sigma	5,16<br>3,88

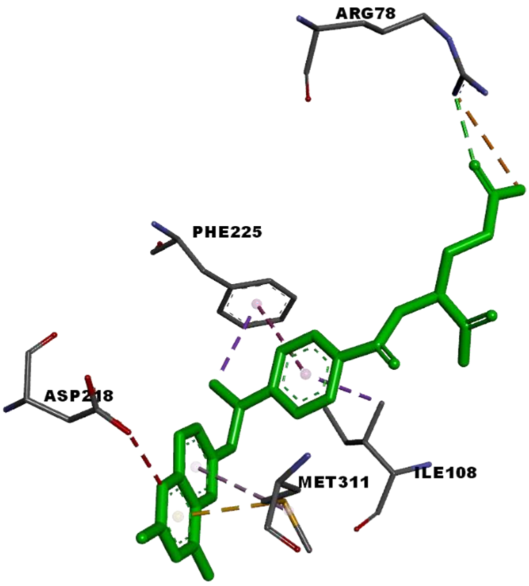

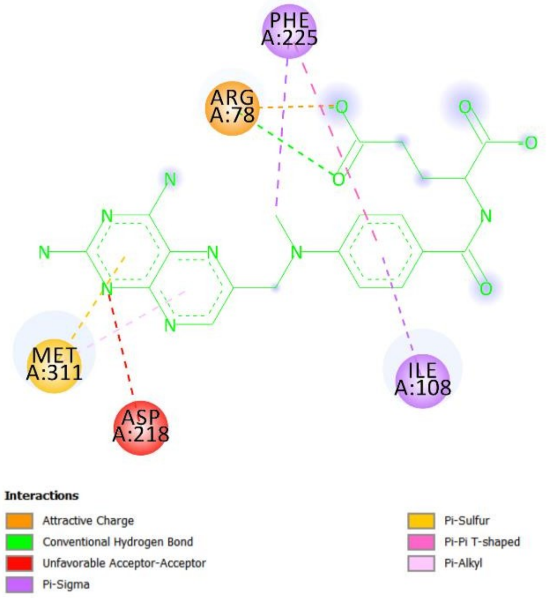

Figure S11: 3D Interactions (5X66-MTX) 2D Interactions (5X66-MTX)

PDB: 5X66
Ligand: Molport-047-399-601 (M601)
Binding affinity: -7,4 Kcal/mol

Table S13: Interactions between amino acid residues of the PDB 5X66 active site with M601.

Residues	Interactions	Bond length (Å)
ASP218	Conventional Hydrogen Bond	2,49
ILE108	Pi-Alkyl	4,90
MET311	Pi-Alkyl	4,58
PHE80	Pi-Donor Hydrogen Bond	2,96
PHE225	Pi-Pi-Stacked	4,18

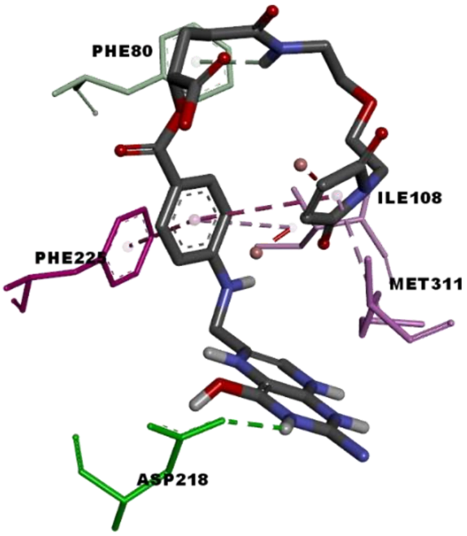

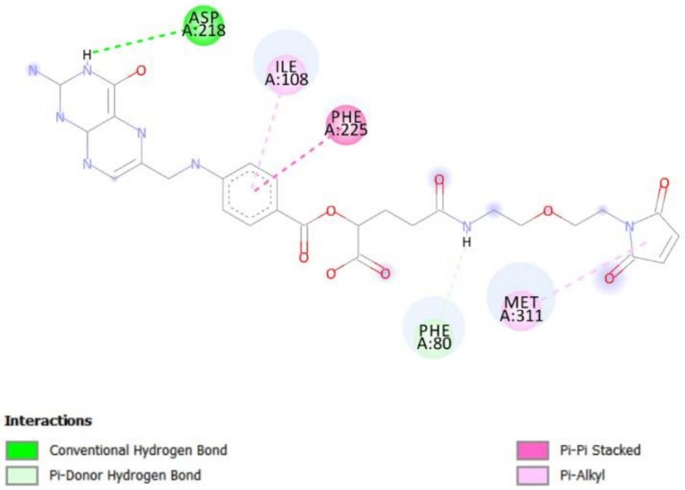

Figure S12: 3D Interactions (5X66-M601) 2D Interactions (5X66-M601)

PDB: 5X66
Ligand: MolPort-008-010-692 (M692)
Binding affinity: -9,1 Kcal/mol

Table S14: Interactions between amino acid residues of the PDB 5X66 active site with M692.

Residues	Interactions	Bond length (Å)
ILE108	Conventional Hydrogen Bond	2,68
	Pi-Sigma	3,79
	Pi-Alkyl	4,35
	Pi-Alkyl	4,82
MET311	Pi-Sulfur	3,88
	Alkyl	4,69
PHE225	Pi-Pi-Stacked	3,78

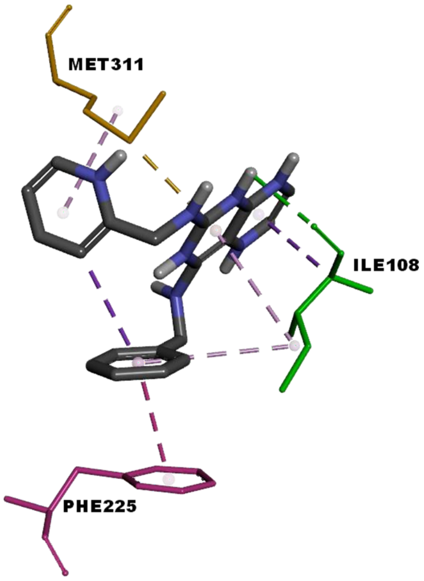

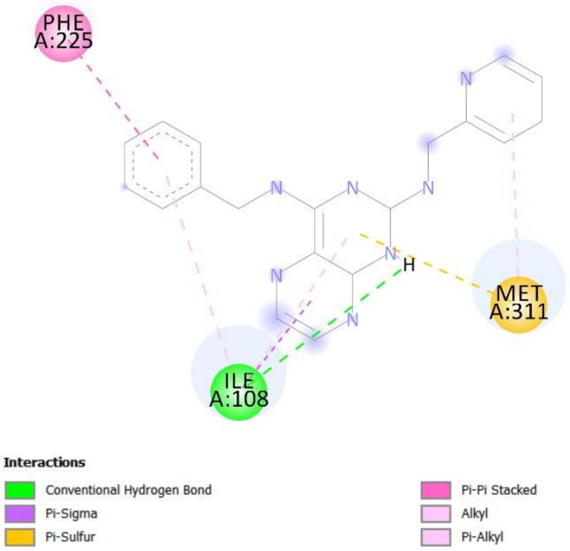

Figure S13: 3D Interactions (5X66-M692) 2D Interactions (5X66-M692)

PDB: 5X66
Ligand: MolPort-008-010-700 (M700)
Binding affinity: -8,1 Kcal/mol

Table S15: Interactions between amino acid residues of the PDB 5X66 active site with M700.

Residues	Interactions	Bond length (Å)
GLU87	Carbon Hydrogen Bond	3,73
ILE108	Pi-Alkyl	4,79
LEU221	Conventional Hydrogen Bond<br>Pi-Alkyl	2,26/2,61<br>4,88
MET311	Pi-Alkyl	5,19
PHE225	Pi-Pi T-Shaped	4,86

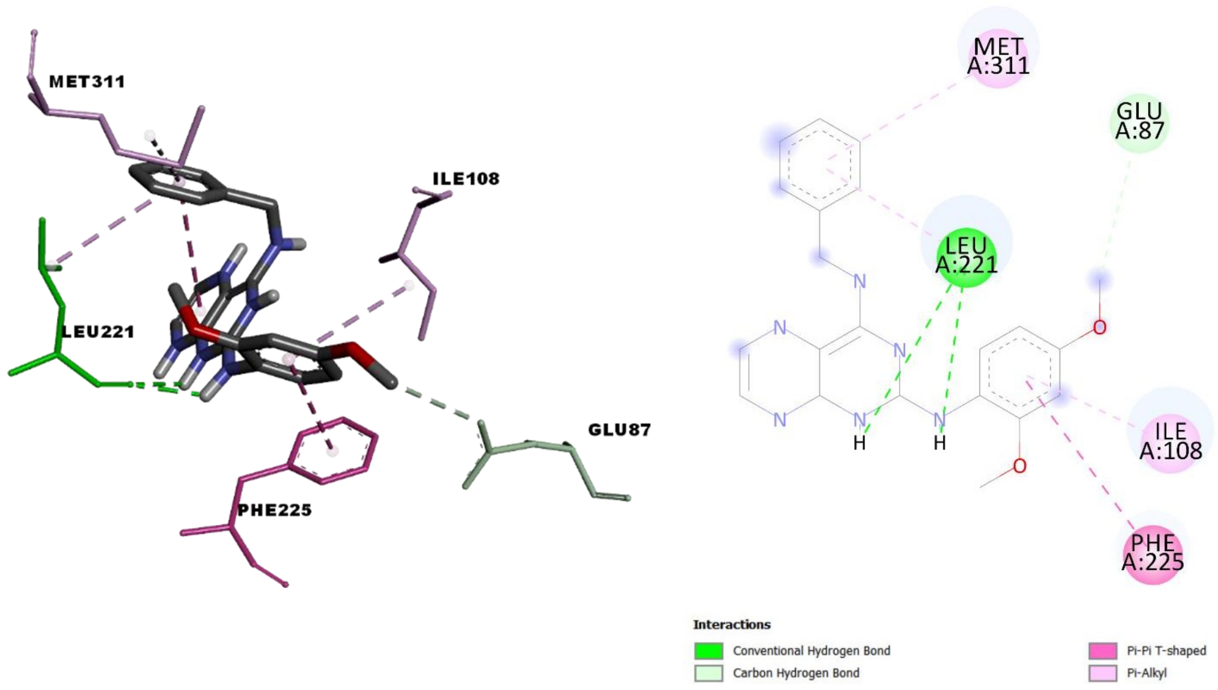

Figure S14: 3D Interactions (5X66-M700) 2D Interactions (5X66-M700)

PDB: 5X66
Ligand: MolPort-008-010-703 (M703)
Binding affinity: -8,1 Kcal/mol

Table S16: Interactions between amino acid residues of the PDB 5X66 active site with M703.

Residues	Interactions	Bond length (Å)
GLU87	Carbon Hydrogen Bond	3,69
ILE108	Pi-Alkyl	4,65
LEU221	Pi-Sigma	3,54/3,58
MET311	Pi-Sulfur	5,36
PHE80	Pi-Pi-Stacked	4,08
PHE225	Pi-Pi-Stacked	4,23

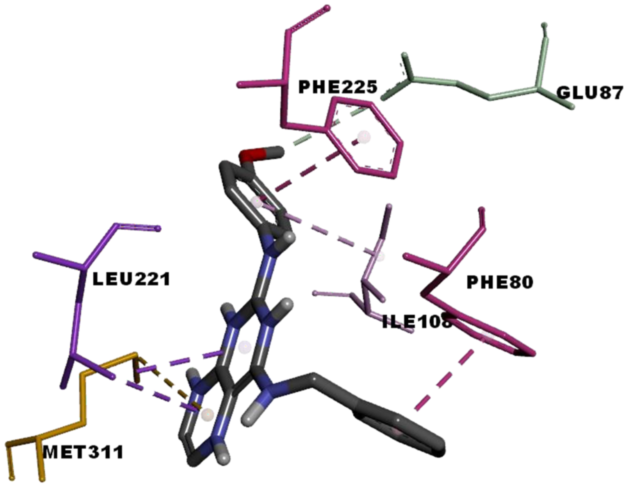

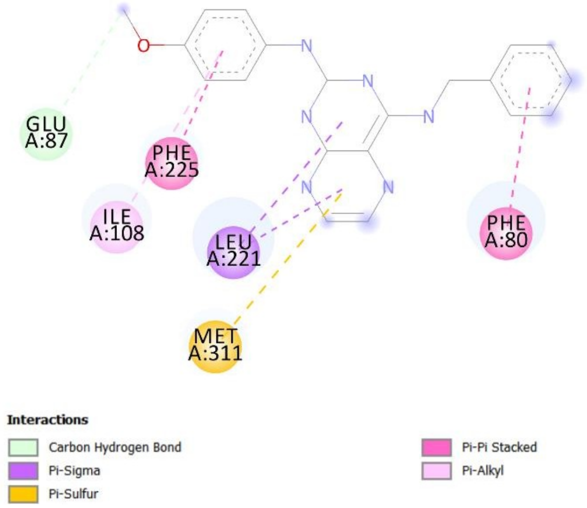

Figure S15: 3D Interactions (5X66-M703) 2D Interactions (5X66-M703)
